# Design, Synthesis, Molecular Modeling, and Biological Evaluation of Novel Thiouracil Derivatives as Potential Antithyroid Agents

**DOI:** 10.3390/molecules23112913

**Published:** 2018-11-08

**Authors:** Samir M. Awad, Yasser M. Zohny, Sahar A. Ali, Shahenda Mahgoub, Ahmed M. Said

**Affiliations:** 1 Pharmaceutical Organic Chemistry Department, Faculty of Pharmacy, Helwan University, Ein-Helwan, Helwan, Cairo 11795, Egypt; samirawad2000@yahoo.com (S.M.A.); dryasserzohny@su.edu.sa (Y.M.Z.); 2 Pharmaceutical Sciences Department, School of Pharmacy, Shaqra University, P.O. Box 11961, Dawadmi 11911, Saudi Arabia; 3 Biochemistry and Molecular Biology Department, Faculty of Pharmacy, Helwan University, Ein-Helwan, Helwan, Cairo 11795, Egypt; ganah_nour@yahoo.com (S.A.A.); shahenda.mahgoub@pharm.helwan.edu.eg (S.M.); 4 Department of Chemistry, University at Buffalo, The State University of New York, Buffalo, NY 14260, USA

**Keywords:** hyperthyroidism, thiouracil, pyrimidine, antithyroid, PTU, LPO

## Abstract

Hyperthyroidism is the result of uncontrolled overproduction of the thyroid hormones. One of the mostly used antithyroid agents is 6-*n-*propyl-2-thiouracil (PTU). The previously solved X-ray crystal structure of the PTU bound to mammalian lactoperoxidase (LPO) reveals that the LPO-PTU binding site is basically a hydrophobic channel. There are two hydrophobic side chains directed towards the oxygen atom in the C-4 position of the thiouracil ring. In the current study, the structural activity relationship (SAR) was performed on the thiouracil nucleus of PTU to target these hydrophobic side chains and gain more favorable interactions and, in return, more antithyroid activity. Most of the designed compounds show superiority over PTU in reducing the mean serum T4 levels of hyperthyroid rats by 3% to 60%. In addition, the effect of these compounds on the levels of serum T3 was found to be comparable to the effect of PTU treatment. The designed compounds in this study showed a promising activity profile in reducing levels of thyroid hormones and follow up experiments will be needed to confirm the use of the designed compounds as new potential antithyroid agents.

## 1. Introduction

Hyperthyroidism is the abnormal excessive production of the T3 and T4 thyroid hormones by the thyroid gland [1,2,3]. This uncontrolled production of thyroid hormones could be due to the presence of autoantibodies (Graves’ disease) that results in overactivation of the thyroid stimulating hormones (TSH) and, subsequently, overproduction of thyroid hormones [4,5,6,7]. In addition, these antibodies stimulate the deiodinases that are responsible for the transformation of prohormone thyroxine (T4) to the triiodothyronine (T3) thyroid hormone. Other causes for the uncontrolled overproduction of thyroid hormones may be inflammation of the thyroid gland or thyroid adenoma [8]. The overproduction of T4 and T3 thyroid hormones can be controlled by either blocking the biosynthesis of the thyroid hormones or by decreasing the conversion of T4 to the active T3 thyroid hormone. These processes could be controlled by specific inhibitors called antithyroid agents [9,10,11,12,13].

Antithyroid agents are relatively simple molecules that are thiourea-based (thionamides) compounds with a thione moiety within a heterocyclic structure. The widely used antithyroid agents are 6-*n*-propyl-2-thiouracil (PTU), methimazole (MMI), 6-methyl-2-thiouracil (MTU), and carbimazole (CBZ) as well as their selenium analogues [14,15,16,17]. The mechanistic details of these agents on how they affect the production of the thyroid hormones are unclear. However, according to the initially proposed mechanism, these agents work by inhibiting the thyroid hormones biosynthesis by interfering with the peroxidase (thyroid peroxidase or lactoperoxidase) mediated iodination of the thyroglobulin tyrosyl residues [18,19,20]. This is usually done by either forming a stable electron donor-acceptor complex with diiodine or coordinating to the metal center of the thyroid peroxidase. The superfamily of mammalian peroxidases consists of four peroxidases: thyroid peroxidase (TPO), lactoperoxidase (LPO), myeloperoxidase (MPO), and eosinophil peroxidase (EPO) [21]. These peroxidases are covalently linked to a heme (iron containing) group and they use hydrogen peroxide (H_2_O_2_) as the electron acceptor, which catalyzes various oxidative reactions. TPO is a transmembrane homodimer that plays an important role in the biosynthesis of the thyroid hormones. The inhibition of TPO seems to be an excellent approach to treat hyperthyroidism. Since 6-*n-*propyl-2-thiouracil or its derivatives can inhibit TPO, a complete understanding of how PTU or its derivatives bind to the TPO catalytic binding site is very important. Although low-resolution crystals of TPO were reported over the years, its structure remains to be determined. The closest known homology of TPO is that of LPO, which shares 48% sequence identity with TPO’s MPO-like domain [22]. In addition, TPO and LPO share similarity in functional properties. This means that the substrate binding sites are high likely to be similar in both TPO and LPO. This was preliminary proven when PTU was tested against LPO and was found to produce a similar effect to that against TPO, which indicates that PTU have similar binding modes with TPO and LPO. The x-ray crystal structure of LPO revealed a substrate-specific binding mode [19,20,21,22,23,24,25,26]. The LPO has its substrate-binding cleft located on the distal heme side. This will be the site where the PTU or any close derivative proposed will bind. In the current study, five different series of thiouracil derivatives were designed, synthesized, and biologically tested (PTU was used as the control drug) to provide more biologically active thiourea-pyrimidine based analogues (Thiouracil) that could be developed further as antithyroid agents. In addition, the binding mode of some analogues to the mammalian LPO was compared to the binding mode of PTU using molecular modeling. The structural differences between the new derivatives and the PTU are changes at positions 4- and 5- of the thiouracil nucleus in the new analogues. In addition, a fused ring (5- or 6- membered) with the pyrimidine ring of the thiouracil was added. These structural changes were performed to investigate how further changes on the thiouracil nucleus could affect the antithyroid activity of these compounds against the excessive secretions of the thyroid hormones T3 and T4 in rats.

## 2. Results and Discussion

### 2.1. Chemistry

**Scheme 1:**
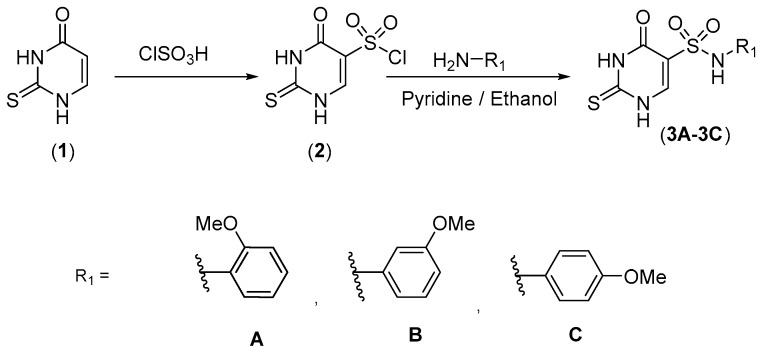
Synthesis of intermediates **3A**–**3C**.

The pyrimidine nucleus itself is unreactive toward electrophilic substitution reactions due to the -I and -M effects of the two nitrogen atoms while 2-thiouracil nucleus (**1**) is active due to the electron releasing effect of -OH and -SH groups [27,28]. Thus, 2-thiouracil could be chlorosulphonated in a good yield, which was reported by Fathalla et al. [29] to give an active sulphonyl chloride (**2**). A positive Lassaigne’s test confirmed this transformation and the appearance of a doublet peak at C6 in its H-NMR spectrum. The sulphonyl chloride (Scheme 1) was reacted with isomers of anisidine (*o*, *m*, and *p*-anisidine) in the presence of a base such as pyridine in ethanol yielding 2-thiouracil-5-sulphonamide derivatives (**3A**–**3C**).

**Scheme 2:**
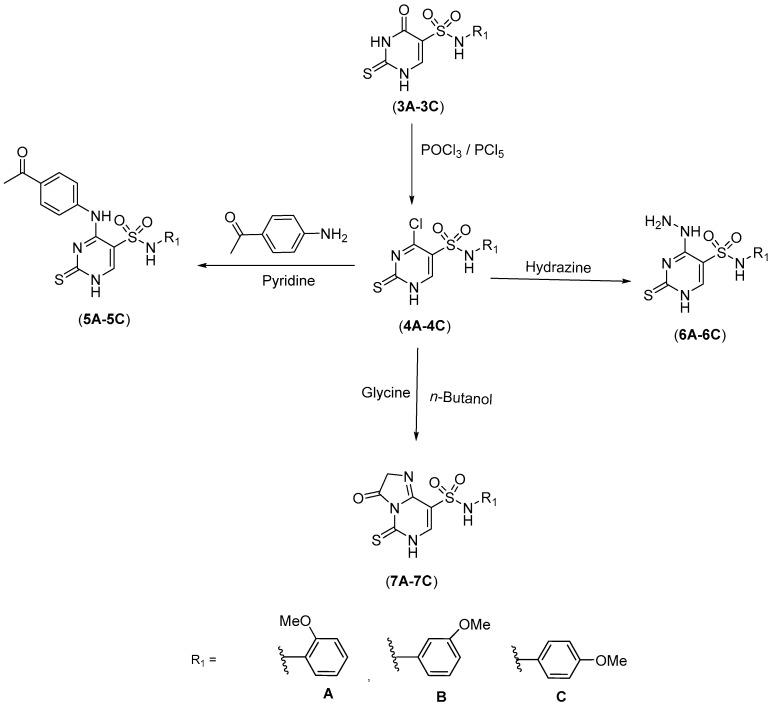
Synthesis of compounds **4A**–**4C**, **5A**–**5C**, **6A**–**6C,** and **7A**–**7C**.

The thiouracil intermediates (**3A**–**3C**) were then chlorinated (Scheme 2) using the POCl_3_/PCl_5_ mixture giving active chloropyrimidine derivatives (**4A**–**4C**). These chloro compounds reacted with *p*-aminoacetophenone in pyridine as a solvent and base yielding aniline compounds **5A**–**5C**. The chlorinated compounds were also hydrazinolysed by NH_2_NH_2_ and gave hydrazinopyrimidines **6A**–**6C**. In another pathway, they were cyclo-condensed with glycine in butanol, which gave imidazopyrimidines (**7A**–**7C**).

**Scheme 3:**
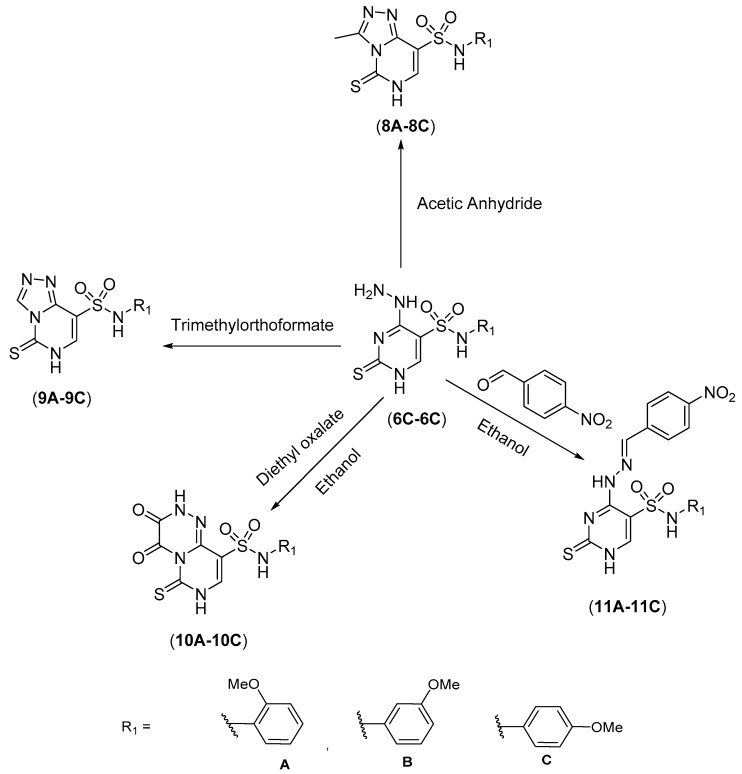
Synthesis of compounds **8A**–**8C**, **9A**–**9C**, **10A**–**10C**, and **11A**–**11C**.

The formed hydrazinopyrimidines (**6A**–**6C**) were used in several pathways. The first pathway had these compounds further cyclo-condensed (Scheme 3) with Ac_2_O into triazolopyrimidines (**8A**–**8C**). The second pathway reacted with the hydrazinopyrimidines with trimethyl orthoformate giving triazolopyrimidines (**9A**–**9C**). The third pathway gave pyrimidotriazines **10A**–**10C** by reaction with diethyl oxalate. The fourth pathway had these compounds condensed with *p*-nitro benzaldehyde and gave the corresponding Schiff’s bases (**11A**–**11C**).

### 2.2. Molecular Modeling and Binding Mode Prediction

**Figure 1:**
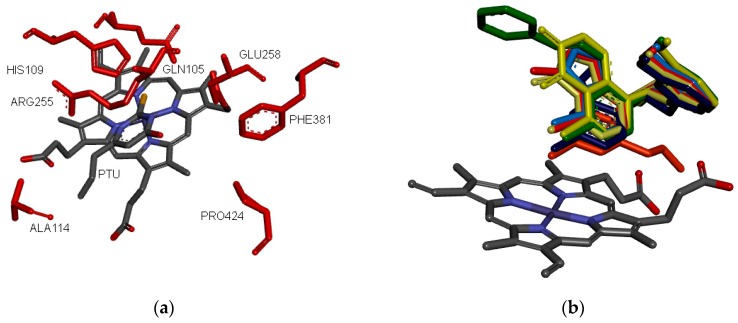
(**a**) Structure of the complex of 6-*n-*propyl-2-thiouracil (PTU) inbound to lactoperoxidase (LPO). (**b**) The overlaying of the modeled structure of inhibitors **6B**, **7B**, **9B**, **10B**, **11B**, and the crystal structure of PTU.

One way to overcome the undesirable state of hyperthyroidism is by inhibiting the function of the mammalian peroxidases TPO and LPO, which catalyze the biosynthesis of thyroid hormones. In a recent study, PTU was shown to have similar IC_50_ values against TPO (30 µM) and LPO (47 µM), respectively [19,20,21,22,23,24,25,26]. This suggests that the PTU binds similarly to both TPO and LPO substrate binding sites. The crystal structure of PTU bound to LPO was recently solved and the binding mode was revealed [22]. To predict and understand the binding mode and the interactions of the designed compounds inside the active site of LPO, the crystal structure (PDB: 5HPW) was used. All the modeling (docking) experiments described here were performed by using the Discovery Studio version 4.5 (Accelrys Inc., San Diego, CA, USA). The required LPO bound to PTU coordinates were downloaded from the Brookhaven website (www.rcsb.org). The hydrogen atoms were then added to both the small molecule and the enzyme. The atom and bond types as well as the protonation state for the small molecule and the binding site residues were checked and corrected when needed. Water molecules were deleted. This was followed by minimizing the complex with the Discovery Studio (DS) force field by using the default parameters. The co-crystal structure (Figure 1a) reveals that PTU binds in the substrate-binding site on the distal heme side of LPO. The orientation of the S-atom of PTU was found to be directed towards the iron center of the heme side. The *n*-propyl hydrophobic side chain on the six-position engages in van der Waals interactions with atoms of neighboring amino acids in the binding site such as Gln 105, His 109, Ala 114, Arg 255, and Glu 258 [23]. These interactions were found to be responsible for the strong binding of PTU to the LPO binding site. The structure of the substrate-binding site where the PTU binds revealed that there are two hydrophobic side chains protruding into the binding site. These side chains are Phe 381 and Pro 424. These side chains were found to be directed towards the O-atom of PTU. As such, the synthesized analogues were designed to target these additional interactions. The modeling studies were performed by using the PTU co-crystal structure with LPO as a starting point and structural changes were performed case by case. The resulting modeled complexes were minimized to convergence by using the DS applied force field. The binding mode for the modeled structures was compared to the binding mode of PTU (Figure 1b) by overlaying the crystal structure of PTU bound to LPO with the modeled structures of the designed compounds. All the modeled structures maintained the same binding mode of PTU.

The designed inhibitors bind similarly to PTU by occupying the hydrophobic channel that is near the heme residue. The binding mode of the designed inhibitors inbound to the LPO binding site are included in the Supplementary Material. It is expected that these compounds will have a similar antithyroid mechanism of action as PTU [25,26]. These compounds, like PTU, are expected to block the synthesis of thyroid hormones by inhibiting the heme containing peroxidases that catalyzes the iodination and coupling of tyrosyl residues on thyroglobulin. In addition, these compounds like PTU could inhibit the iodothyronine deiodinase (ID-1) and, thereby, block the conversion of thyroxine (T4) to 3,3′,5-triiodothyronine (T3) within the thyroid and in peripheral tissues. It should be noted that the designed compounds as well as PTU will only inhibit the formation of new thyroid hormones and will not remove thyroid hormones, which are already in the thyroid or in the blood stream. Further investigation and experiments will be performed in the future to determine the exact antithyroid mechanism of action of these compounds and determine any direct or indirect antithyroid effect for these compounds.

### 2.3. Biological Screening

**Figure 2:**
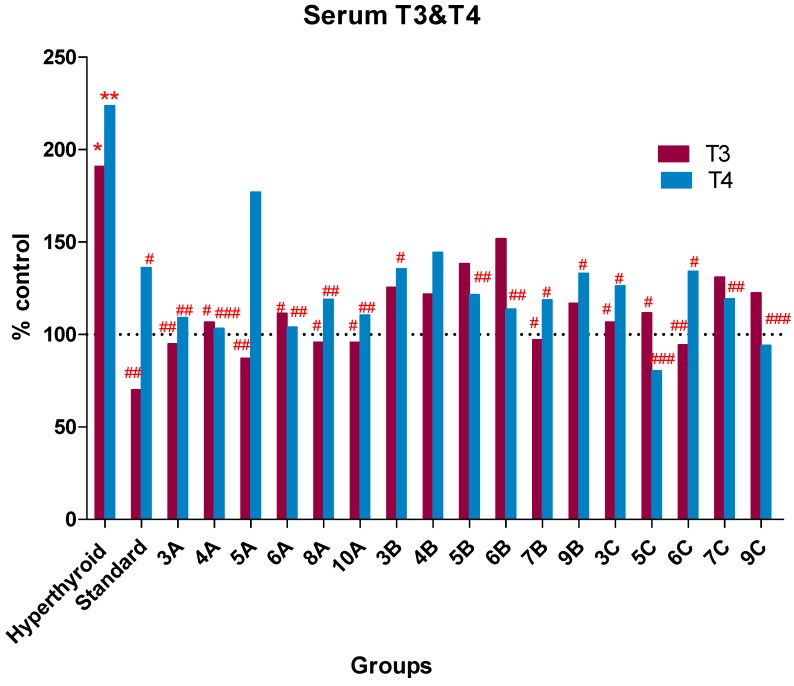
Effect of antithyroid derivatives on the mean serum levels of T3 and T4 in rats. Data are presented as the percentage in the control group. *: significant from the control group at *p* < 0.05. **: significant from the control group at *p* < 0.01 and 0.001. #, ## and ###: significant from Hyperthyroidism group at *p* < 0.05, 0.01 and 0.001, respectively.

The synthesized thiouracil derivatives were evaluated for their antihyperthyroid activity by using a thyroxine-induced hyperthyroid model [30]. The antithyroid activity was obtained by comparing the mean serum T3 and T4 levels in hyperthyroid rats after treatment by using the synthesized thiouracil derivatives as well as the standard PTU. The synthesized compounds (10 mg/kg) were assessed for their anti-thyroid activity using the thyroxine-induced hyperthyroid model, which was comparable to PTU used as the standard antithyroid drug when comparing the mean serum T3 and T4 levels between the different studied groups. Administration of Thyroxine (600 µg/kg) to euthyroid animals significantly increased mean serum levels of T3 and T4 hormones. These results matched with what was observed earlier [31,32,33,34,35]. All rat groups treated with the designed inhibitors (10 mg/kg) showed a significant decrease in the mean serum T4 levels in the hyperthyroid model (except for drugs **5A** and **4B**) compared to the hyperthyroid untreated group, which is depicted in Table 1. Moreover, derivatives **3A**, **4A**, **5A**, **8A**, **10A**, **7B**, **3C**, and **6C** also showed a comparable decrease in the mean serum level of T3 compared to the hyperthyroid untreated group. Comparing the antithyroid activity of these compounds to that of the reference antithyroid drug PTU showed that nearly all the synthesized derivatives showed a significant decrease in the mean serum T4 level (except for drugs **5A** and **4B**), which is shown in Figure 2. However, they were not able to reach this significant reduction in the mean T3 level when compared to PTU. Previous studies [34,35] showed that patients treated with thionamides take three to eight weeks to become euthyroid since they block new hormone synthesis and any already formed thyroxine T4 and T3 stored in the colloid must be secreted and metabolized for clinical improvement to occur. Thus, antithyroid drugs typically reduce levels of both T3 and T4, but levels of T3 may take longer to return to normal. There are several possible explanations for the development of simultaneously high serum T3 and low serum T4 levels in patients with hyperthyroid disease during or after treatment. Iodide organification is impaired by thionamide antithyroid drugs. The resulting increase in iodide leakage from the gland creates a state of intrathyroidal iodine deficiency, which favors T3 production. Furthermore, hyperthyroidism is associated with increased T4 5′-deiodinase activity in both the thyroid gland and extrathyroidal tissues, which leads to increased T4 to T3 conversion. This agrees with the study results, which showed that the designed compounds reduced the serum T4 more than T3 levels in hyperthyroid rats.

**Figure 3:**
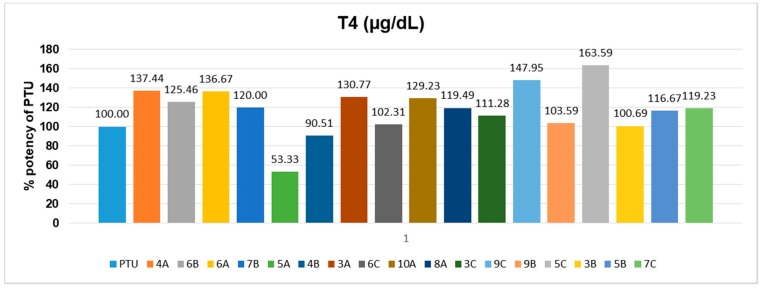

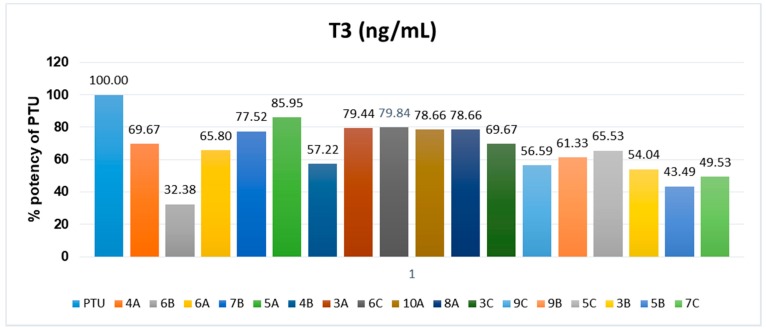
Potency of antithyroid derivatives compared to 6-*n-*propyl-2-thiouracil (PTU).

As shown in Figure 2 and Figure 3, the effect of the designed inhibitors on the mean serum level of T4 is significantly more potent (3% to 60% more) than the effect exerted by PTU except drugs **5A** and **4B**, which suggests an anti-thyroid role for these derivatives. However, only drugs (**3A**, **4A**, **5A**, **8A**, **10A**, **7B**, **3C**, and **6C** showed a comparable decrease in the mean serum level of T3 (Figure 3) compared to the hyperthyroid untreated group. In addition, lower potency was compared to the standard PTU drug. Our observations, therefore, revealed that the antithyroid effect produced by the treatment of the hyperthyroid rats with the thiouracil derivatives for 14 days was more significant on serum T4 levels than serum T3 levels.

In addition, SAR strategies performed in this study involved replacement of =O (C4) with –Cl, which is shown in compounds **4A**–**4C**. This strategy slightly improved activity against T4 hormone levels (comparing **3A** to **4A**, 5% improvement). However, the activity against T3 hormone levels was not improved. Our second strategy was to replace =O with a *p-*acetyl aniline side chain as in compounds **5A**–**5C**. This strategy significantly improved the activity of the compounds against T4 hormone levels (Comparing **3C** to **5C**, 36% improvement). The activity against T3 hormone levels was also maintained. Our third strategy was to replace =O with the -NHNH_2_ group. This strategy-maintained activity against both T3 and T4 hormones levels (compare **3A** to **6A**). The final strategy was to form a fused ring (five or six membered) to the thiouracil ring. This strategy somewhat maintains activity against T3 and T4 hormone levels.

In general, the data of selected pyrimidine derivatives **3A**, **4A**, **5A**, **6A**, **7A**, **8A**, **9A**, **10A**, and **11A** showed that 2-thiouracil-5-sulphonamide is active. When chlorinated, it is still active, but when chlorine is replaced by the NH group, the activity is reduced such as in compounds **5A**, **6A**, and **11A**. Cyclo-condensation of chloro derivative **4A** retains the activity such as in compounds **7A**, **8A**, **9A**, and **10A**.

## 3. Materials and Methods

### 3.1. General Experimental Methods

All melting points were measured by using an Electrothermal IA 9100 apparatus (Shimadzu, Kyoto, Japan). IR spectra were recorded as potassium bromide pellets on a Perkin-Elmer 1650 spectrophotometer (Perkin-Elmer, Boston, MA, USA). ^1^H-NMR spectra (300MHz) were recorded in dimethyl sulfoxide (DMSO) by employing tetramethyl silane (TMS) as an internal standard on Varian Mercury 300 MHz NMR Spectrometer (Varian, Crawley, West Sussex, UK) and the chemical shifts (δ) were expressed as ppm against TMS as an internal standard. Electron impact mass spectra (EI) were recorded on a Shimadzu QP–2010 instrument (Shimadzu, Japan) at 70 eV. Microanalyses were operated by using the vario, Elementar apparatus (Shimadzu, Japan). Thin layer Chromatography plates (TLC) (Merck, Kenilworth, NJ, USA) was used to monitor the progress of all the reactions using chloroform-methanol (3:1) as a mobile phase and spots were visualized by iodine vapors or by irradiation with UV-light (254 nm).

### 3.2. Synthesis of N-(2,3 or 4-Methoxyphenyl)-4-oxo-2-thioxo-1,2,3,4-tetrahydropyrimidine-5-sulphonamides (***3A**–**3C***)

A mixture of 2-thiouracil-5-sulphonamide **2** (1.13 g, 0.005 mol), isomers of anisidine (0.6 g, 0.005 mol), and pyridine (0.4 mL, 0.005 mol) in absolute ethanol (50 mL) was heated under reflux for 12 h and then cooled, filtered off, dried, and recrystallized from dimethyl formamide (DMF)/water.

Yield, **3A** (0.8 g, 67%), m.p. 273 °C to 275 °C. **3B** (0.6 g, 61%), m.p. 256 °C to 258 °C. **3C** (0.5 g, 58%), m.p. 282–284 °C. IR (KBr): 3300–3020 (3 NH, very broad), 1660 (CO), 1320, 1140 (SO_2_) cm^−1^. ^1^H-NMR (DMSO-*d*_6_): δ 4.1 (s, 3H, OCH_3_), 7.1–7.3 (m, 4H, ArH), 8.2 (s, 1H, pyrimidine), 9.8, 10.2, 10.5 (s, 3NH, D_2_O-exchangeable). MS (EI) *m*/*z* (10.43%) 313.12 [M^+^]. Analysis for C, H, and N, C_11_H_11_N_3_O_4_S_2_ Calcd: C, 42.17, H, 3.51, N, 13.42. Found: C, 42.31, H, 3.7, N, 13.55.

### 3.3. Synthesis of 4-Chloro-N-(2,3 or 4-methoxyphenyl)-2-thioxo-1,2-dihydropyrimidine-5-sulphonamide (***4A**–**4C***)

A mixture of **3A**–**3C** (0.01 mol) and phosphorus pentachloride (0.01 mol) in phosphorus oxychloride (20 mL) was heated on a steam bath for 3 h and the reaction mixture was poured gradually on crushed ice. The precipitate was filtered off, dried, and then crystallized from DMF/water.

Yield, **4A** (0.6 g, 61%), m.p. 283–284 °C. **4B** (0.7 g, 64%), m.p. 267–268 °C. **4C** (0.8 g, 69%), m.p. 262–264 °C. IR (KBr): 3310–3026 (3 NH, very broad), 1670 (CO), 1325, 1135 (SO_2_) cm^−1^. ^1^H-NMR (DMSO-*d*_6_): δ 4.2 (s, 3H, OCH_3_), 7.1–7.2 (m, 4H, ArH), 8.1 (s, 1H, pyrimidine), 9.7, 10.6 (s, 2NH, D_2_O-exchangeable). MS (EI) *m*/*z* (1.74%) 331.57 [M^+^], (0.57%) [M + 2H]^+^, Analysis for C, H, and N, C_11_H_10_N_3_O_3_S_2_Cl, Calcd: C, 39.82, H, 3.02, N, 12.67. Found: C, 39.71, H, 3.29, N, 12.45.

### 3.4. Synthesis of 4-(4-acetylphenyl) amino]-N-(2,3or4-methoxyphenyl)-2-thioxo-1,2-dihydropyrimidine-5-sulphonamides (***5A**–**5C***)

A mixture of **4A**–**AC** (1 mol) and *p-*aminoacetophenone (1 mol) in pyridine (30 mL) was refluxed for 8 to 10 h and the reaction mixture poured gradually on water and then neutralized until acidification. The precipitate was filtered off, dried, and then crystallized from DMF/water.

Yield, **5A** (0.7 g, 66%), m.p. 243–245 °C. **5B** (0.8 g, 73%), m.p. 286 to 288 °C. **5C** (0.8 g, 65%), m.p. 277–279 °C. IR(KBr): 3315–3028 (3 NH, very broad), 1670 (CO), 1690 (CO) 1322,1145 (SO_2_) cm^−1^. ^1^H-NMR (DMSO-*d*_6_): δ 2.2 (s, 3H, CH_3_), 4.2 (s, 3H, OCH_3_), 7.2–7.9 (m, 8H, ArH), 8.3 (s, 1H, pyrimidine), 6.7, 9.6, 10.6 (s, 3NH, D_2_O-exchangeable). MS (EI) *m*/*z* (9.85%) 430.32 [M^+^]. Analysis for C, H, and N, C_19_H_18_N_4_O_4_S_2_ Calcd: C, 53.02, H, 4.19, N, 13.02. Found: C, 53.19, H, 3.99, N, 13.42.

### 3.5. Synthesis of 4-Hydrazinyl-N-(2,3 or 4-methoxyphenyl)-2-thioxo-1,2-dihydropyrimidine-5-sulphonamides (***6A**–**6C***)

A mixture of **4A**–**4C** (0.01 mol) and hydrazine hydrate (0.01 mol) in methanol (10 mL) was stirred for 8 h. The precipitate was filtered off, dried, and then crystallized from DMF/water.

Yield, **6A** (0.5 g, 55%), m.p. 263 °C to 265 °C. **6B** (0.6 g, 62%), m.p. 294 °C to 296 °C. **6C** (0.8 g, 68%), m.p. 280 °C to 282 °C. IR(KBr): 3340–3044 (3NH, NH_2_ very broad), 1681 (CO), 1320, 1148 (SO_2_) cm^−1^. ^1^H-NMR (DMSO-*d*_6_): δ, 4.1 (s, 3H, OCH_3_), 7.2–7.4 (m, 4H, ArH), 8.1 (s, 1H, pyrimidine), 6.7, 9.6, 10.6, 10.4 (s, NH_2_, 3NH, D_2_O-exchangeable). MS (EI) *m*/*z* (18.45%) 325.13 [M^+^]. Analysis for C, H, and N, C_11_H_13_N_5_O_3_S_2_, Calcd: C, 40.62, H, 3.39, N, 21.54. Found: C, 40.47.19, H, 3.41, N, 21.42.

### 3.6. Synthesis of N-(2,3 or 4-methoxyphenyl)-3-oxo-5-thioxo-1,2,3,5,6,8a-hexahydroimidazo[1,2-c] pyrimidine-8-sulphonamides (***7A**–**7C***)

A mixture of **4A**–**4C** (0.01 mol) and glycine (0.01 mol) in *n*-butanol (30 mL) was heated under reflux for 3 h. The solid separated was refluxed with anhydrous acetic acid (5 mL) for 2 h. The precipitate was filtered off, dried, and then crystallized from DMF/water.

Yield, **7A** (0.6 g, 57%), m.p. 243–245 °C. **7B** (0.7 g, 64%), m.p. 267 °C to 269 °C. **7C** (0.8 g, 66%), m.p. 287°C to 289 °C. IR (KBr): 3345–3034 (3 NH, very broad), 1687 (CO), 1320, 1140 (SO_2_) cm^−1^.^1^H-NMR (DMSO-*d*_6_): δ 2.1(s, 2H, CH_2_), 4.2 (s, 3H, OCH_3_), 7.1–7.4 (m, 4H, ArH), 8.2 (s, 1H, pyrimidine), 9.7, 10.4, 10.5 (s, 3NH, D_2_O-exchangeable). MS (EI) *m*/*z* (11.45%) 352.08 [M^+^]. Analysis for C, H and N, C_13_H_12_N_4_O_4_S_2_ Calcd: C, 44.32, H, 3.41, N, 15.91. Found: C, 44.19, H, 3.48, N, 16.02.

### 3.7. Synthesis of N-(2,3 or 4-methoxyphenyl)-3-methyl-5-thioxo-5,6-dihydro [1,2,4] triazolo[4,3-c] pyrimidine-8-sulphonamides (***8A**–**8C***)

A mixture of **6A**–**6C** (0.01 mol) and acetic anhydride (30 mL) was heated under reflux for 4 h. The solid obtained was filtered off, dried, and crystallized from DMF/water.

Yield, **8A** (0.6 g, 58%), m.p. 277 °C to 279 °C. **8B** (0.7 g, 68%), m.p. 244 °C to 246 °C. **8C** (0.8 g, 67%), m.p. 276 °C to 278 °C. IR (KBr): 3350–3037 (2NH, very broad), 1320, 1140 (SO_2_) cm^−1^. ^1^H-NMR (DMSO-*d*_6_): δ 2.3(s, 3H, CH_3_), 4.1 (s, 3H, OCH_3_), 7.1–7.4 (m, 4H, ArH), 8.1 (s, 1H, pyrimidine), 10.2, 10.5 (s, 2NH, D_2_O-exchangeable). MS (EI) *m*/*z* (11.45%) 352.08 [M^+^]. Analysis for C, H, and N, C_13_H_13_N_5_O_3_S_2_, Calcd: C, 44.44, H, 3.70, N, 19.94. Found: C, 44.19, H, 3.68, N, 19.77.

### 3.8. Synthesis of 8-(N-(2,3 or 4-methoxyphenyl)-5-thioxo-5,6-dihydro [1,2,4] triazolo [4,3-c] pyrimidine-8-sulphonamides (***9A**–**9C***)

A mixture of **6A**–**6C** (0.01 mol) and trimethyl orthoformate (30 mL) was heated under reflux for 6 h. The solid obtained was filtered off, dried, and crystallized from DMF/water.

Yield, **9A** (0.6 g, 58%), m.p. 283 to 285 °C. **9B** (0.8 g, 69%), m.p. 276 to 278 °C. **9C** (0.8 g, 68%), m.p. 297 to 299 °C. IR (KBr): 3335–3027 (2NH, very broad), 1320, 1140 (SO_2_) cm^−1^. ^1^H-NMR (DMSO-*d*_6_): δ 2.1(s, 1H, CH), 4.1 (s, 3H, OCH_3_), 7.2–7.4 (m, 4H, ArH), 8.1 (s, 1H, pyrimidine), 10.2, 10.4 (s, 2NH, D_2_O-exchangeable). MS (EI) *m*/*z* (11.45%) 337.24 [M^+^]. Analysis for C, H, and N, C_12_H_11_N_5_O_3_S_2_, Calcd: C, 42.73, H, 3.26, N, 20.77. Found: C, 42.87, H, 3.38, N, 20.67.

### 3.9. Synthesis of N-(2,3 or 4-methoxyphenyl)-3,4-dioxo-6-thioxo-3,4,6,7-tetrahydro-2H-pyrimido[6,1-c] [1,2,4] triazine-5-sulphonamides (***10A**–**10C***)

A solution of **6A**–**6C** (0.01 mol) and diethyl oxalate (0.01 mol) in absolute ethanol (40 mL) was heated under reflux for 12 h. The solid obtained was filtered off, dried, and crystallized from DMF/water.

Yield, **10A** (0.5 g, 52%), m.p. 267 to 269 °C. **10B** (0.6 g, 64%), m.p. 256 to 258 °C. **10C** (0.6 g, 66%), m.p. 277 to 279 °C. IR (KBr): 3322–3037 (2NH, very broad), 1680, 1687 (2 CO), 1320, 1140 (SO_2_) cm^−1^. ^1^H-NMR (DMSO-*d*_6_): δ 4.1 (s, 3H, OCH_3_), 7.2–7.5 (m, 4H, ArH), 8.2 (s, 1H, pyrimidine), 6.8 10.2, 10.3 (s, 3NH, D_2_O-exchangeable). MS (EI) *m*/*z* (1.38%) 381.24 [M^+^]. Analysis for C, H, and N, C_13_H_11_N_5_O_5_S_2_, Calcd: C, 40.94, H, 2.89, N, 18.37. Found: C, 40.87, H, 2.99, N, 18.45.

### 3.10. Synthesis of N-(2,3 or 4-Methoxyphenyl)-4-[(2E)-2-(4-nitrobenzylidene) hydrazinyl]-2-thioxo-1,2,3,4-tetrahydropyrimidine-5-ulphonamides (***11A**–**11C***)

A mixture of **6A**–**6C** (0.01 mol) and *p-*nitro benzaldehyde (0.01 mol) in ethanol (30 mL) was heated under reflux for 8 h. The solid obtained was filtered off, dried, and crystallized from DMF/water.

Yield, **11A** (0.7 g, 70%), m.p. 257 to 259 °C. **11B** (0.5 g, 54%), m.p. 277 to 278 °C. **11C** (0.8 g, 78%), m.p. 258 to 259 °C. IR (KBr): 3332–3027 (3NH, very broad), 1320, 1140 (SO_2_) cm^−1^. ^1^H-NMR (DMSO-*d*_6_): δ 4.1 (s, 3H, OCH_3_), 6.8 (s, 1H), 7.2–7.4 (m, 8H, ArH), 8.2 (s, 1H, pyrimidine), 6.9, 10.3, 10.5 (s, 3NH, D_2_O-exchangeable). MS (EI) *m*/*z* (0.837%) 460.12 [M^+^]. Analysis for C, H, and N, C_18_H_16_N_6_O_5_S_2_, Calcd: C, 46.96, H, 3.48, N, 18.26. Found: C, 46.85, H, 3.56, N, 18.33.

### 3.11. Animals

The complete course of the experiment was conducted by using male Wistar albino rats (200–250 g), reared and maintained in the animal house of the institution, and provided free access to pelleted food and water ad libitum. The rats were maintained in a controlled environment (12 h light and dark cycle) for about a week for acclimatization. The animal ethics committee of the Faculty of Pharmacy, Helwan University (1 October 2016) approved the protocol of the study. The study was conducted in accordance with the EC, directive 86/609/EEC for animal experiments [32]. (Ethical code number: 0011A-16; Date: 6 December 2016) [32]. After the course of treatment, the rats were still alive and showed no signs of toxicity.

### 3.12. Induction of Hyperthyroidism

Hyperthyroidism was induced in experimental rats by administrating Thyroxine (600 µg/kg) orally for 14 days, which was previously reported [33] and induction of hyperthyroidism was confirmed by analyzing the serum thyroid hormone levels. Propylthiouracil (PTU (10 mg/kg)) was used as a standard antithyroid drug and administered orally in compliance with literature experiments [34,35]. Equivalent doses from the selected thiouracil derivatives were given orally to the corresponding rat groups. Prior to the main study, a pilot study was done on a few numbers of rats of different groups to compare the changes of their thyroid gland weight (details are in the Supplementary Material) that is assumed to reflect the alteration in thyroid status. Comparison showed no significant difference in the mean weight of thyroid gland between the groups used.

### 3.13. Experimental Design

One hundred and forty rats (20 groups of 7 rats each) were available to investigate the antithyroid effect of the selected thiouracil derivatives. These rat groups were divided as follows: Group 1 was the normal control and Group 2 included the hyperthyroid induced rats (Thyroxine (600 µg/kg)), which served as a positive control group. Group 3 included hyperthyroid induced rats (Thyroxine (600 µg/kg)) treated with a standard drug (PTU (10 mg/kg)). As in group 3, the hyperthyroid induced rats were used in groups 4–20 (thiouracil derivatives used are **3A**, **4A**, **5A**, **6A**, **8A**, **10A**, **3B**, **4B**, **5B**, **6B**,**7B**, **9B**, **3C**, **5C**, **6C**, **7C**, and **9C**, resp.). The treated groups administered PTU and different derivatives orally for 14 days. For each group, body weights were determined, and blood was collected by a retro-orbital puncture under light ether anesthesia. Serum was separated by centrifugation at 3000 rpm for 10 min and serum produced was used for the biochemical analysis. Serum T3 and T4 levels were analyzed by using Mouse/Rat Triiodothyronine (T3) and Mouse/Rat Thyroxine (T4) ELISA Kits (Sigma-Aldrich Co., St Louis, MO, USA).

### 3.14. Data Presentation and Statistical Analysis

The data were represented as mean ± SEM. Significant differences between groups were tested by using GraphPad InStat software version 3.05 (GraphPad Inc., La Jolla, CA, USA). Appropriate graphs were plotted when needed using GraphPad Prism version 5 for Windows (GraphPad Inc., USA). A *P* value less than 0.05 was considered statistically significant.

## 4. Conclusions

In this study, we have utilized medicinal chemistry coupled with structure-based design strategies to identify more potent thiouracil derivatives that can lower the levels of T4 and T3 thyroid hormones in hyperthyroid rats more than what the standard treatment using PTU can do. The data revealed that we have managed to establish a SAR by doing several modifications to the thiouracil nucleus. All the designed inhibitors significantly reduced the mean serum level of T4 more than the PTU treated group except for compounds **5A** and **4B**. In addition, inhibitors **3A**, **4A**, **5A**, **6A**, **8A**, **10A**, **7B**, **3C**, **5C**, and **6C** showed a comparable effect in decreasing the mean serum level of T3 to that of PTU.

## Figures and Tables

**Table 1:** Effect of various treatments on the mean serum levels of T3 and T4 in rats.

Groups	Serum T3 (ng/mL)	Serum T4 (µg/dL)
Control	60.5 ± 5.08	4.45 ± 1.1
Hyperthyroid group	115.5 ± 22.47 *	9.96 ± 1.28 **
Standard (PTU 10 mg/kg)	42.4 ± 1.44 ^##^	6.06 ± 0.58 ^#^
Compound **3A**	57.43 ± 1.43 ^##^	4.86 ± 0.48 ^##^
Compound **4A**	64.57 ± 3.96 ^#^	4.60 ± 0.24 ^###^
Compound **5A**	52.67 ± 2.42 ^##^	7.88 ± 0.29
Compound **6A**	67.4 ± 1.86 ^#^	4.63 ± 0.67 ^##^
Compound **8A**	58 ± 3.72 ^#^	5.3 ± 0.4 ^##^
Compound **10A**	58 ± 4.16 ^#^	4.92 ± 0.28 ^##^
Compound **3B**	76 ± 2.77	6.03 ± 0.36 ^#^
Compound **4B**	73.67 ± 3.43	6.43 ±1.25
Compound **5B**	83.71 ± 4.61	5.41 ± 0.51 ^##^
Compound **6B**	91.83 ± 6.84	5.06 ± 0.502 ^##^
Compound **7B**	58.83 ± 3.49 ^#^	5.28 ± 0.63 ^#^
Compound **9B**	70.67 ± 3.35	5.92 ± 0.61 ^#^
Compound **3C**	64.57 ± 1.94 ^#^	5.62 ± 0.83 ^#^
Compound **5C**	67.6 ± 3.91 ^#^	3.58 ± 0.35 ^###^
Compound **6C**	57.14 ± 2.52 ^##^	5.97 ± 0.69 ^#^
Compound **7C**	79.29 ± 4.79	5.31 ± 0.49 ^##^
Compound **9C**	74.13 ± 3.91	4.19 ± 0.61 ^###^

*: significant from the control group at *p* < 0.05. **: significant from the control group at *p* < 0.01 and 0.001. ^#^, ^##^ and ^###^: significant from hyperthyroidism group at *p* < 0.05, 0.01 and 0.001, respectively.

## Supplementary Materials

The supplementary materials are available online. Supplementary data includes molecular modeling and binding mode evaluation to representative inhibitors as well as their spectral data.

## Abbreviations

PTU	6-*n-*propyl-2-thiouracil
LPO	mammalian lactoperoxidase
TPO	thyroid peroxidase
T4	prohormone thyroxine
T3	triiodothyronine thyroid hormone
POCl_3_	phosphorus oxychloride
PCl_5_	Phosphorus pentachloride
Ac_2_O	Acetic anhydride
