# Supplementary material for: Design, Synthesis, Molecular Modeling, and Biological Evaluation of Novel Thiouracil Derivatives as Potential Antithyroid Agents
Source: Molecules. 2018 Nov 8;23(11):2913. doi: 10.3390/molecules23112913 (PMC6278332; doi:10.3390/molecules23112913)
Supplement: Supplementary file 1 [file molecules-23-02913-s001.pdf]

Supplementary Information

Design, Synthesis, Molecular Modeling, and Biological Evaluation of Novel Thiouracil Derivatives as Potential Antithyroid Agents

Samir M. Awad[1], Yasser M. Zohny[1,2], Sahar A. Ali[3], Shahenda Mahgoub[3] and Ahmed M. Said [1,4*]

[1] Pharmaceutical Organic Chemistry Department, Faculty of Pharmacy, Helwan University, Ein-Helwan, Helwan, Cairo 11795, Egypt; samirawad2000@yahoo.com (S.M.A.); dryasserzohny@su.edu.sa (Y.M.Z.); ahmedmoh@buffalo.edu (A.M.S)
[2] Pharmaceutical sciences department, School of Pharmacy, Shaqra University 11961, Dawadmi 11911, Kingdom of Saudi Arabia
[3] Biochemistry and Molecular Biology Department, Faculty of Pharmacy, Helwan University, Ein-Helwan, Helwan, Cairo 11795, Egypt; ganah_nour@yahoo.com (S.A.A.); shahenda.mahgoub@pharm.helwan.edu.eg (S.M.)
[4] Department of Chemistry, University at Buffalo, The State University of New York, Buffalo, NY 14260, United States
* Correspondence: ahmedmoh@buffalo.edu; Tel.: +1716-907-5016

Table of Content:

1. Molecular Modeling and Binding mode of representative Inhibitors…………S2
2. Thyroid gland dissection……………………………………………..……..S6
3. Representative spectra……………………………..…………………...…...S8

1. Molecular modeling and Binding mode prediction of Representative Inhibitors:

1.1 Binding mode of **6B**:

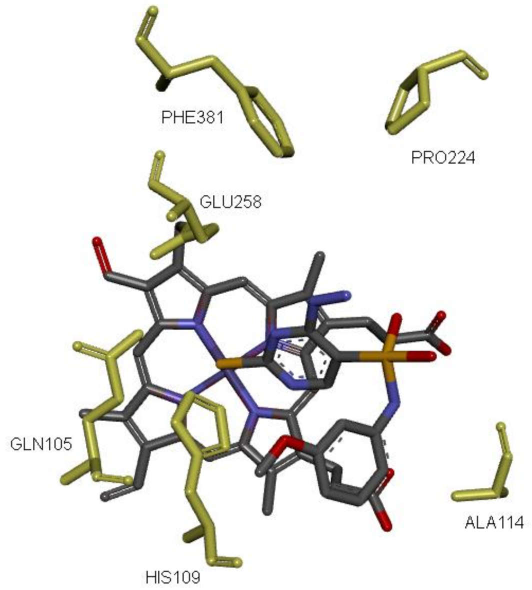

Figure 1. Expected binding mode of compound **6B** inside the LPO active site.

1.2 Binding mode of **7B**:

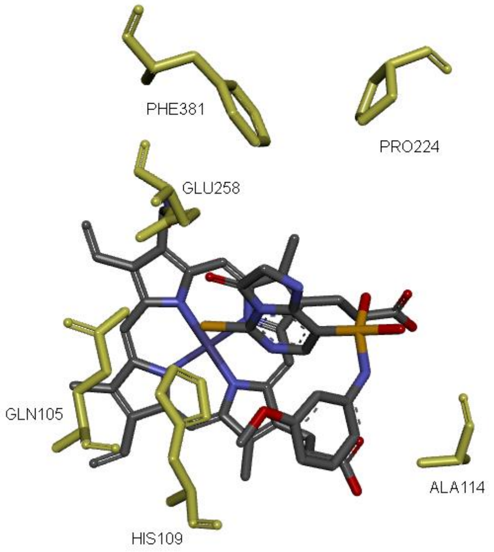

Figure 2. Expected binding mode of compound **7B** inside the LPO active site.

2.4. Binding mode of **9B**:

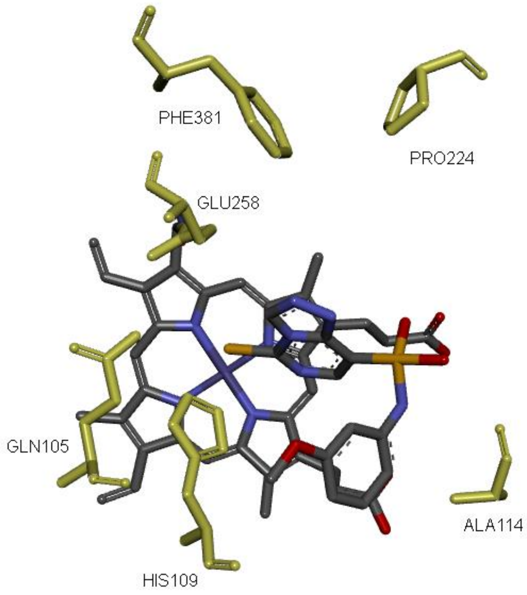

Figure 3. Expected binding mode of compound **9B** inside the LPO active site.

2.5. Binding mode of **10B**:

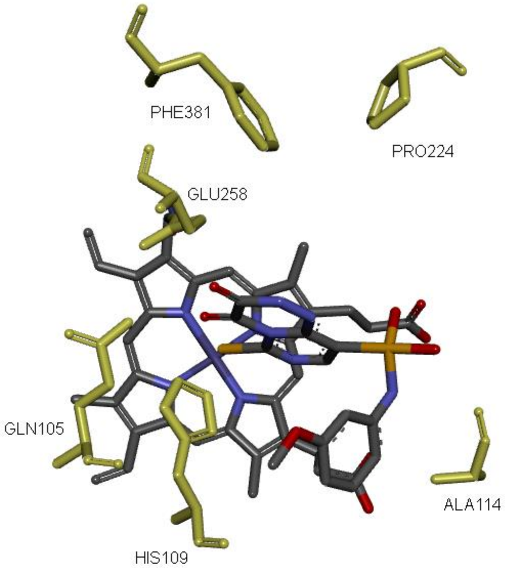

Figure 4. Expected binding mode of compound **10B** inside the LPO active site.

2.6. Binding mode of **11B**:

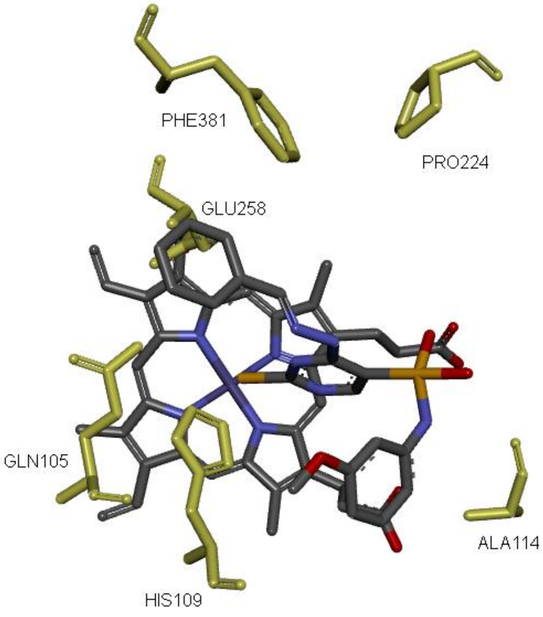

Figure 5. Expected binding mode of compound **11B** inside the LPO active site.

2. **Thyroid gland dissection for one of the treated rats:**

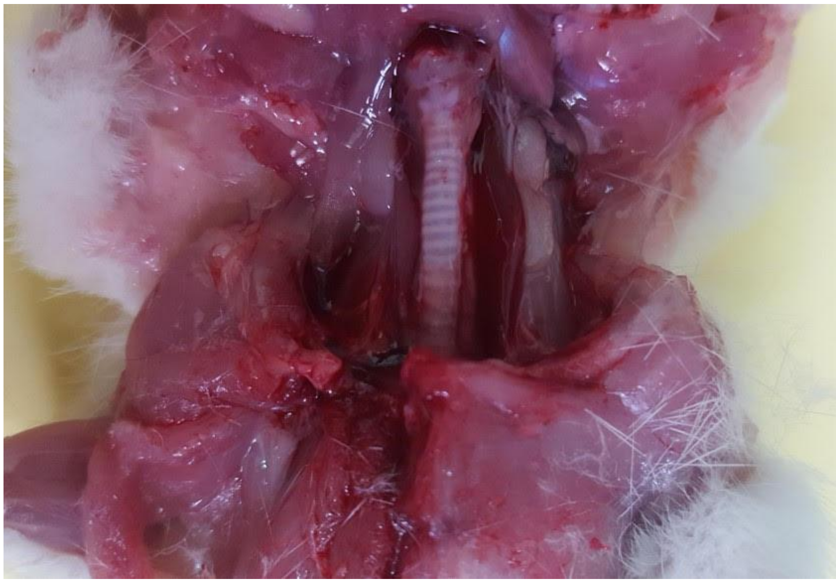

Figure 6. Dissected rat thyroid gland showing the change in thyroid gland size after treatment with Compound **3A**.

The following results are for the pilot study considering Thyroid gland weights. The process of thyroid gland dissection (Figure 6) is time consuming, and for 140 rats will take long time, so we did this piolet study. 4 Groups were chosen (control, Hyperthyroid, PTU treated and Compound 3A treated groups), 3 rats each, and the dissection for the thyroid gland was performed. There were no significant differences between the thyroid gland weights among the 4 groups using the one-way analysis of variance (ANOVA).

Table 1. Mean values of thyroid gland weights in control, hyperthyroid, PTU & compound 3A groups.

Group	Number of rats	Thyroid Weight (gm)	SEM
**Control**	3	0.01987	8.819×10^{-5}
**Hyperthyroid group**	3	0.02097	0.0004910
**PTU treated group**	3	0.01990	5.774×10^{-5}
**Compound 3A treated group**	3	0.02023	0.0002028

Data is expressed as mean ± SEM, P = 0.0671

SEM is standard error of the mean.

Table 2. Effect of the studied drugs and PTU on the body weight of hyperthyroid rats .

Group	Body weight (gm)
Control	197.48± 7.617
HT	173.00± 3.306*
PTU	184.83±8.419
3A	198.48± 7.494#
4A	192.05±6.850
5A	178.05±4.988
6A	198.67±8.787#
8A	181.81±6.421
10A	200.52± 5.938#
3B	193.28± 6.853
4B	192.29± 2.715#
5B	203.91±7.901#
6B	197.38±6.446#

7B	177.43 ± 7.261
9B	191.72 ± 3.142
3C	194.81 ± 4.193
5C	201.19 ± 5.906##
6C	187.67 ± 6.766
7C	199.90 ± 10.151
9C	199.47 ± 7.778#

Data are expressed as mean ± SEM

*significant from control group at p<0.05

#,## significant from hyperthyroid group at p<0.05 and p<0.01, respectively

The mean body weight was compared among the different rat groups at the end of the experiment as shown in Table 2. The hyperthyroid group showed significant decrease in the body weight of the rats compared to control group (p<0.05). The group treated with PTU gained more weight than the hyperthyroid group but did not reach significant. Treatment with different synthesized drugs markedly improved the growth performance (except for drug 7B). Yet, only the groups treated with drugs (3A, 6A, 10A, 4B, 5B, 6B, 5C, 9C showed significant weight increment compared to hyperthyroid group (P<0.05).

3. Representative spectra:

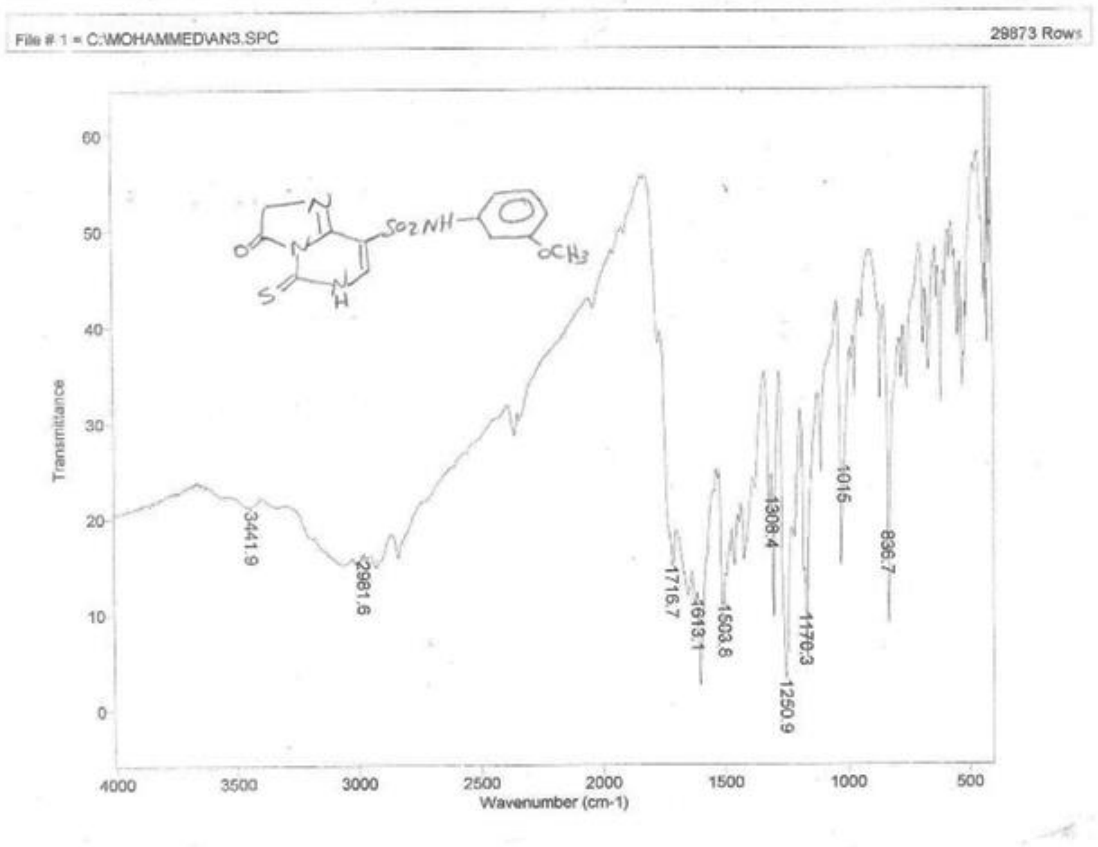

Figure 7. IR spectrum of compound **7B.**

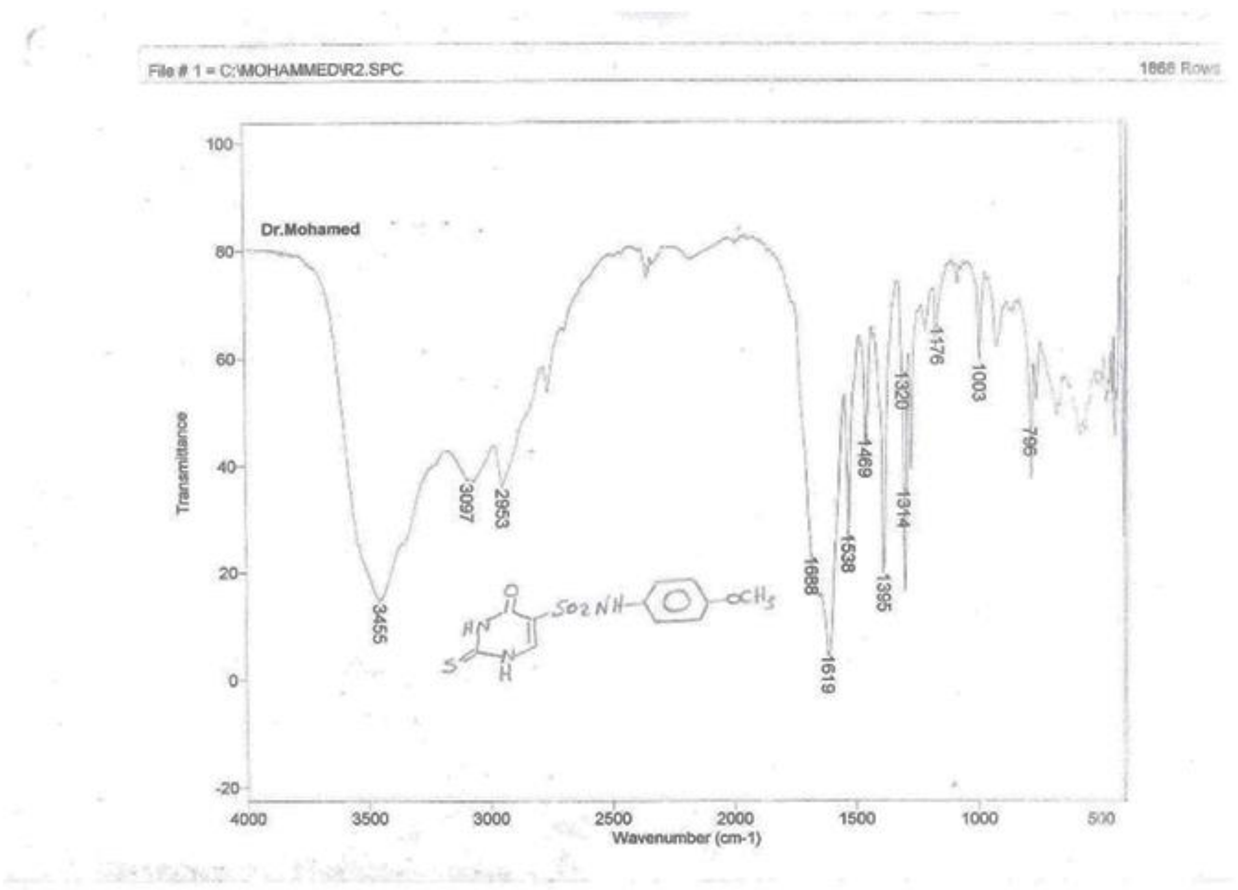

Figure 8. IR spectrum of compound **3C**.

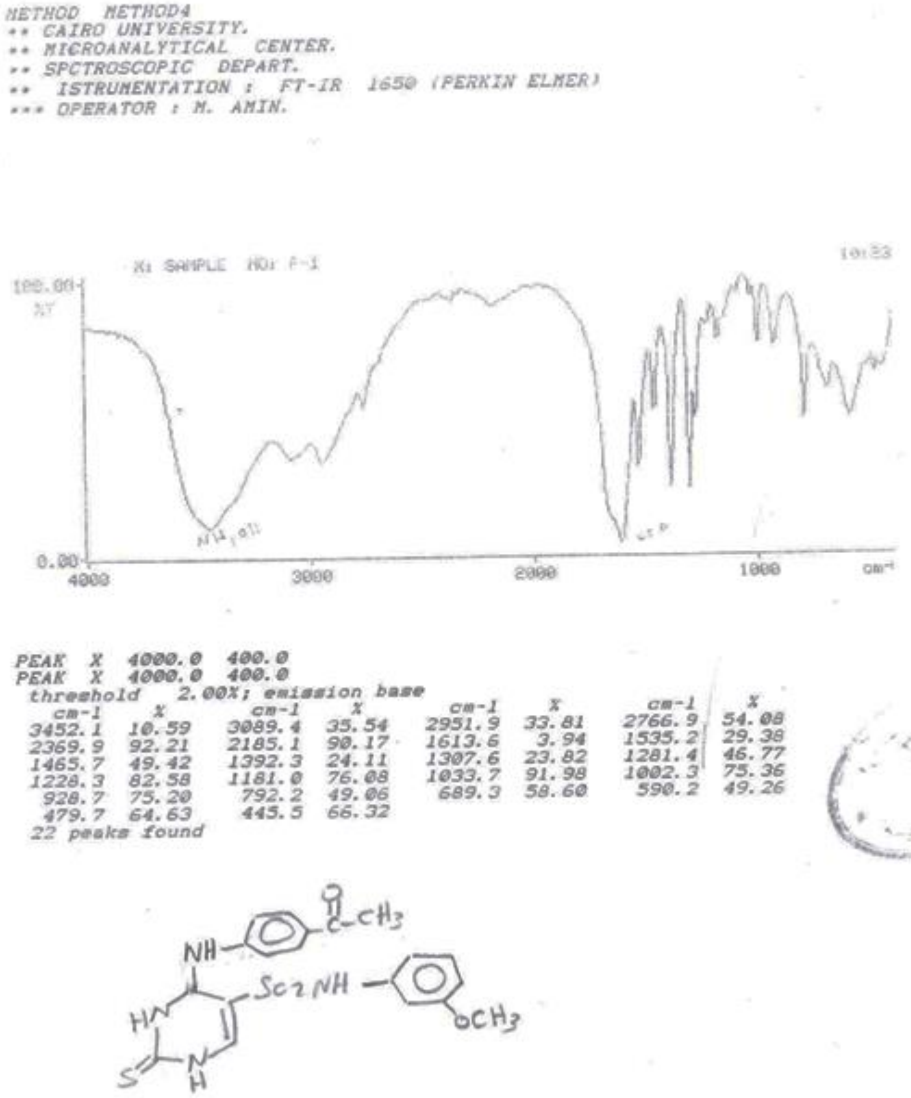

Figure 9. IR spectrum of compound **5B.**

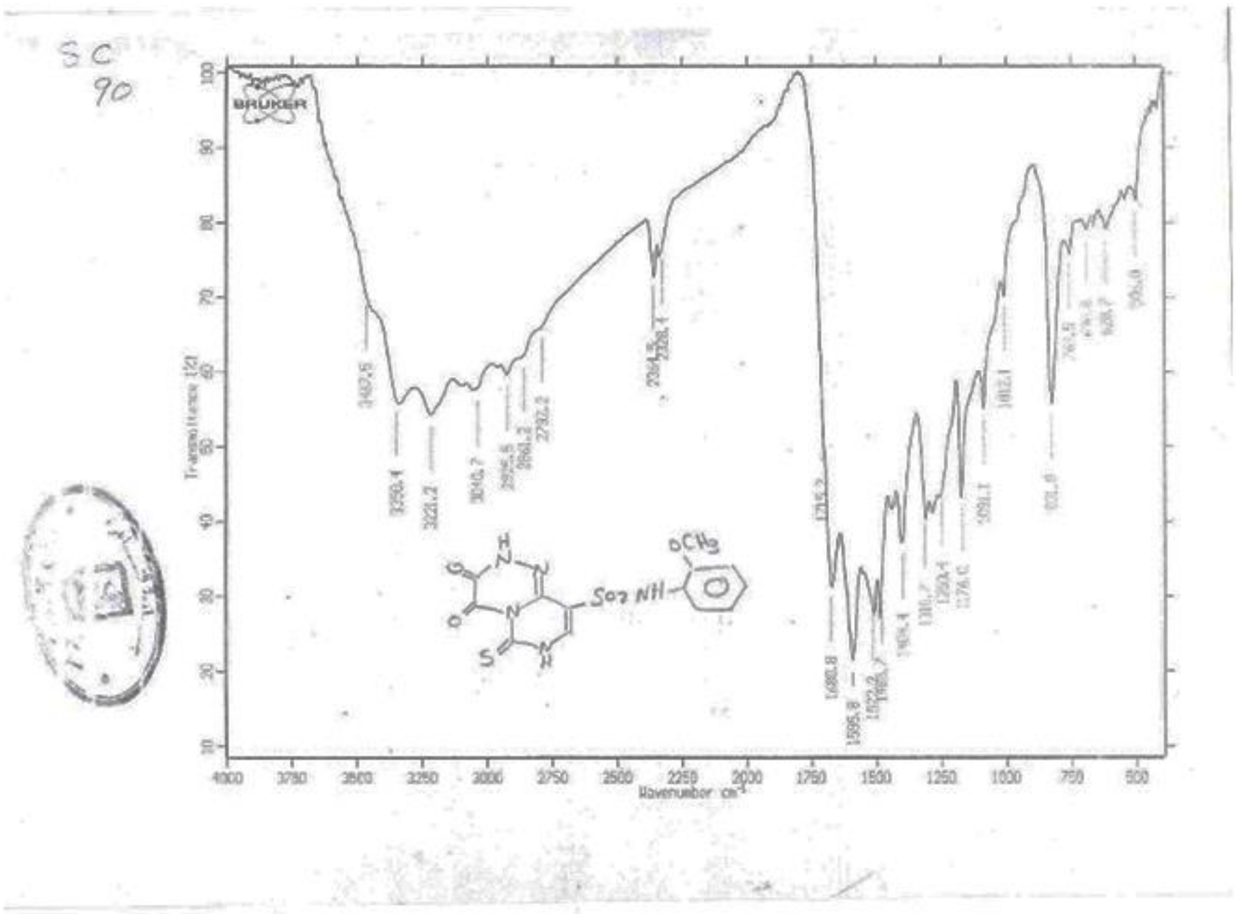

Figure 10. IR spectrum of compound **10A**.

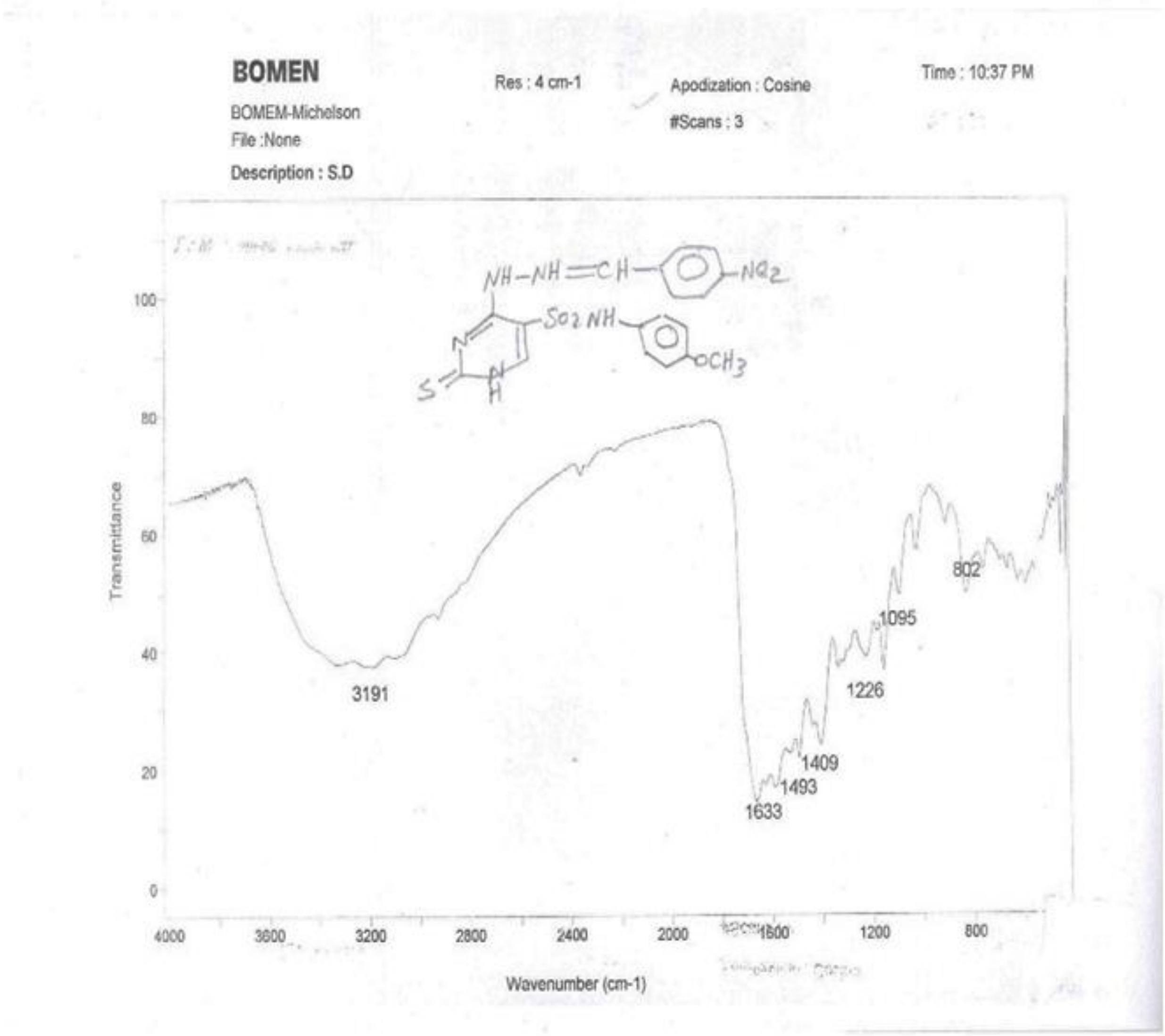

Figure 11. IR spectrum of compound **11C**.

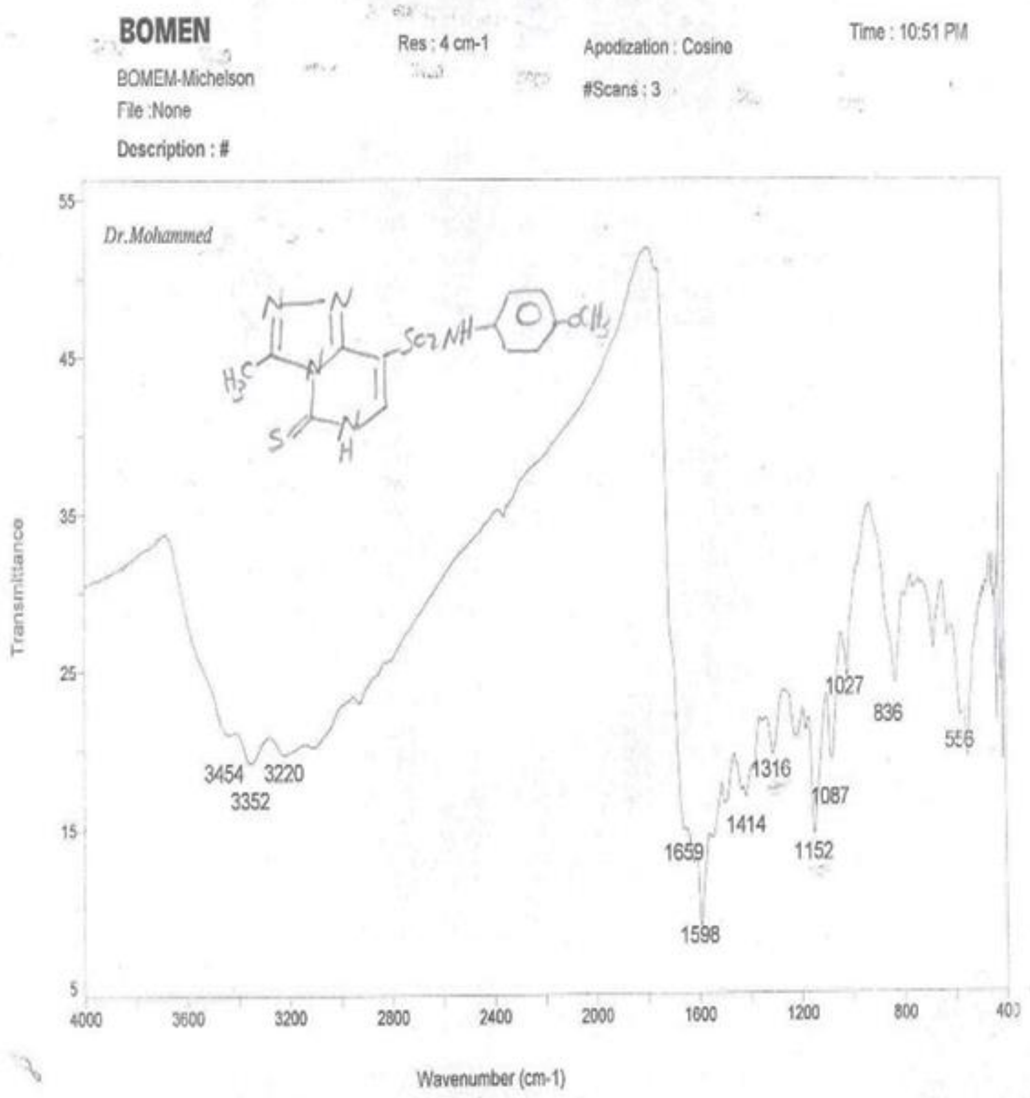

Figure 12. IR spectrum of compound **8C**.

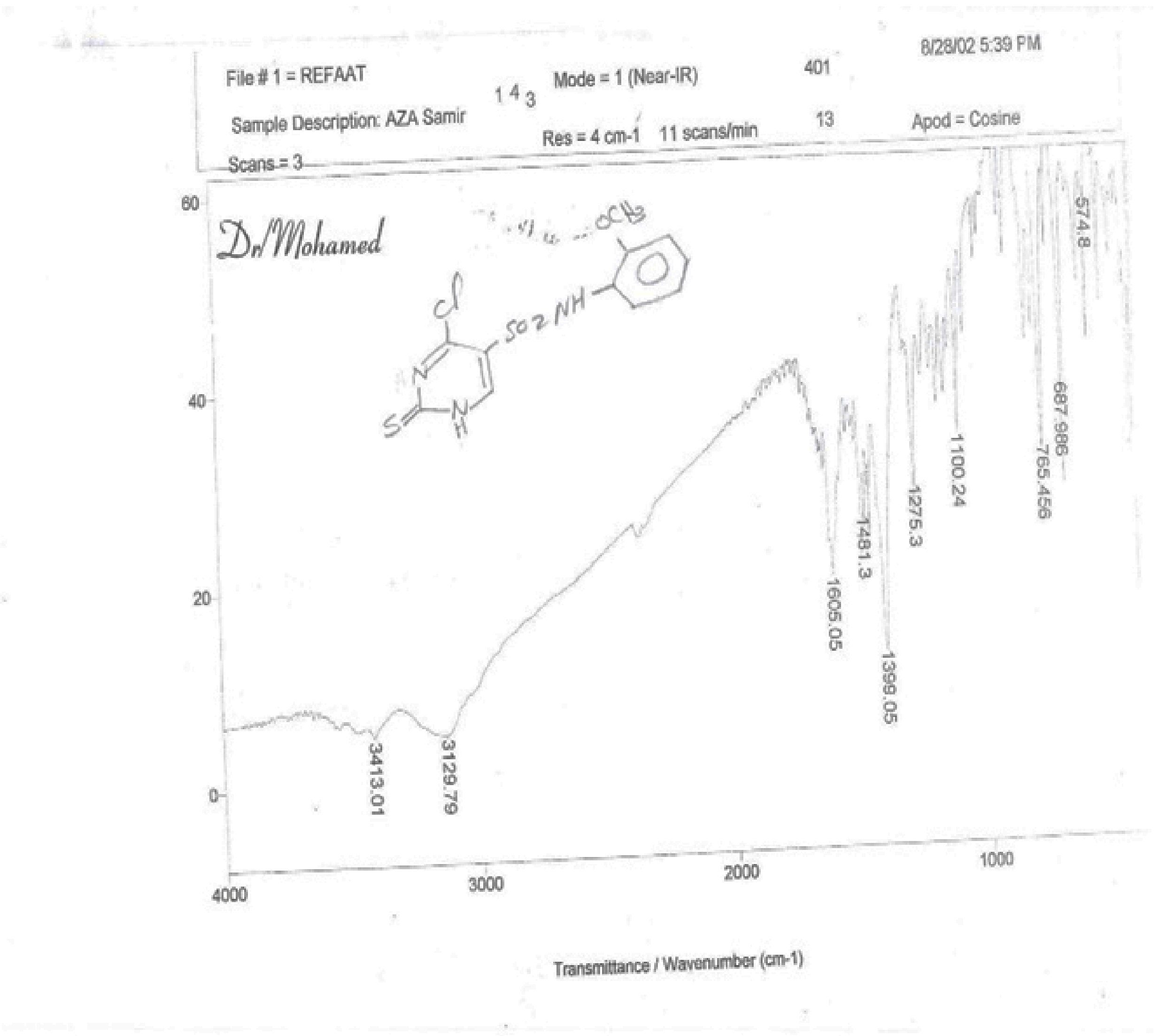

Figure 13. IR spectrum of compound **4A**.

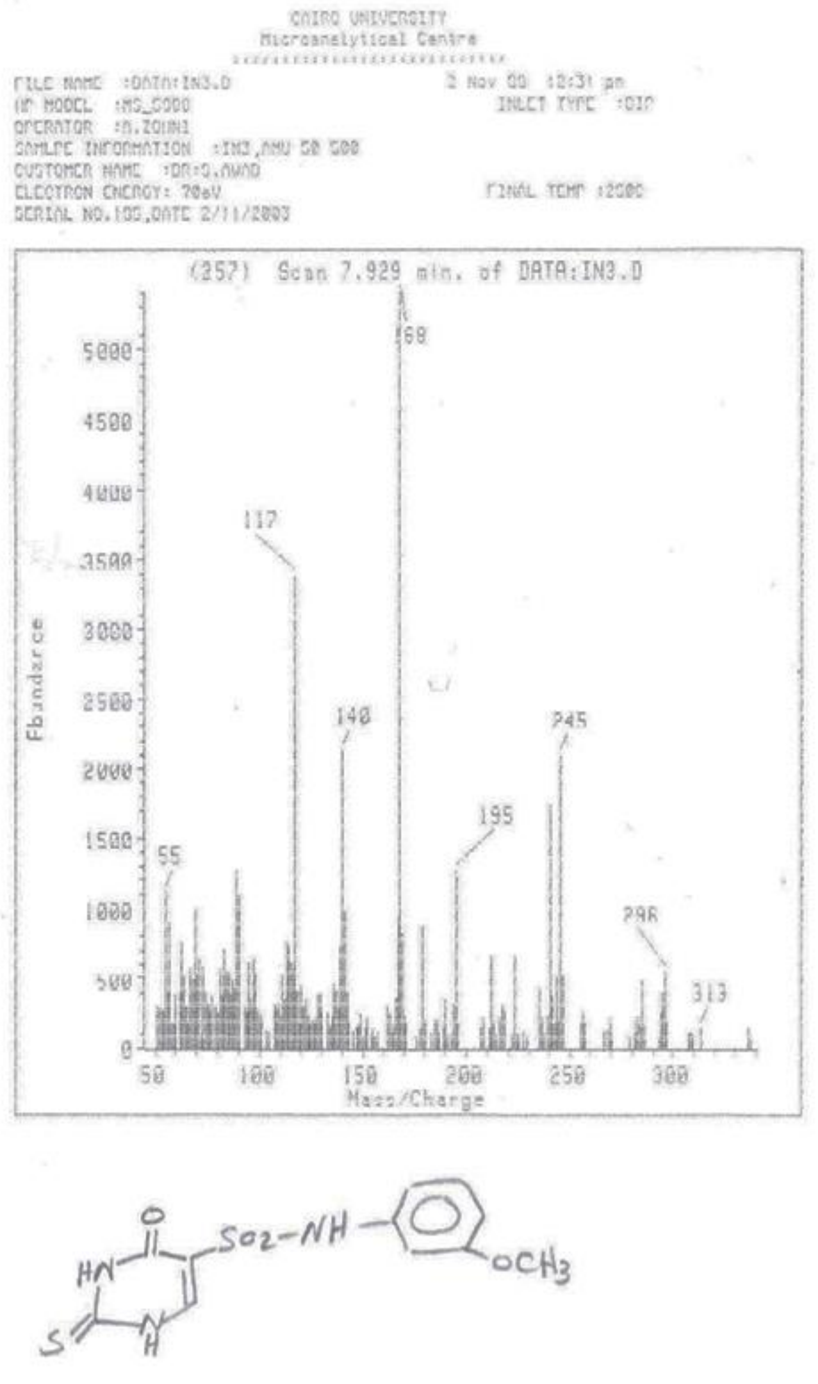

Figure 14. Low resolution mass spectrum of compound **3B**.

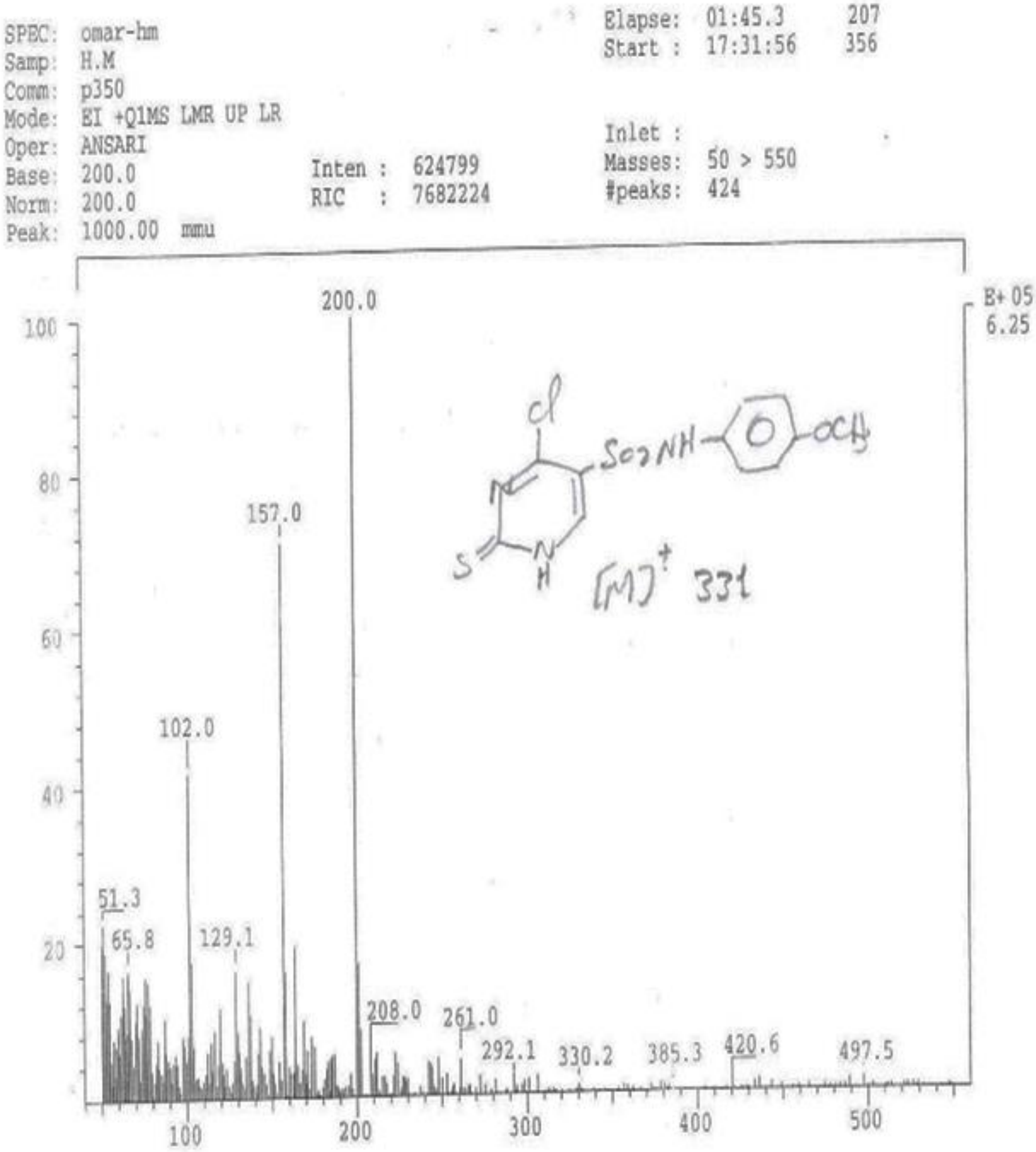

Figure 15. Low resolution mass spectrum of compound **4C.**

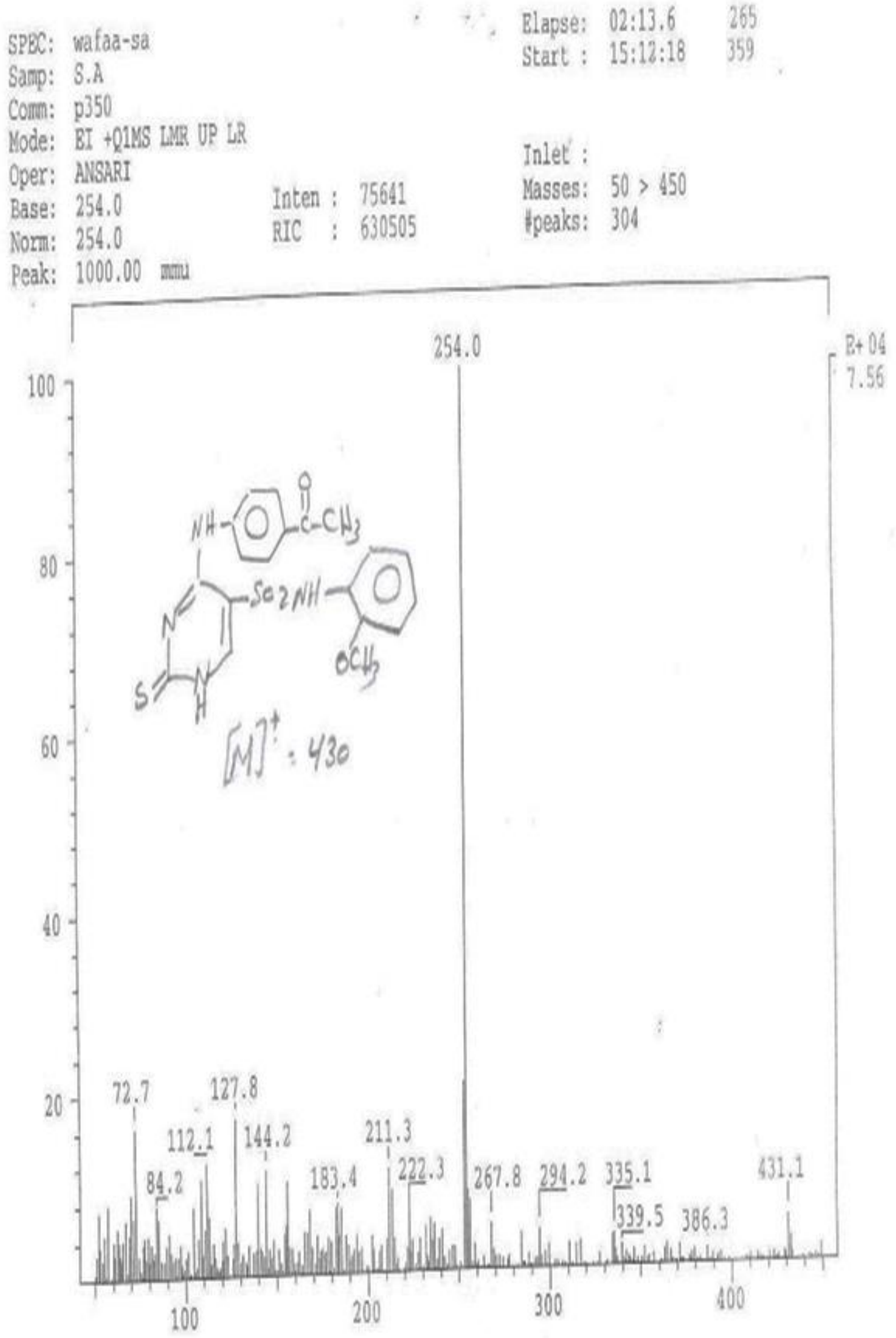

Figure 16. Low resolution mass spectrum of compound **5A.**

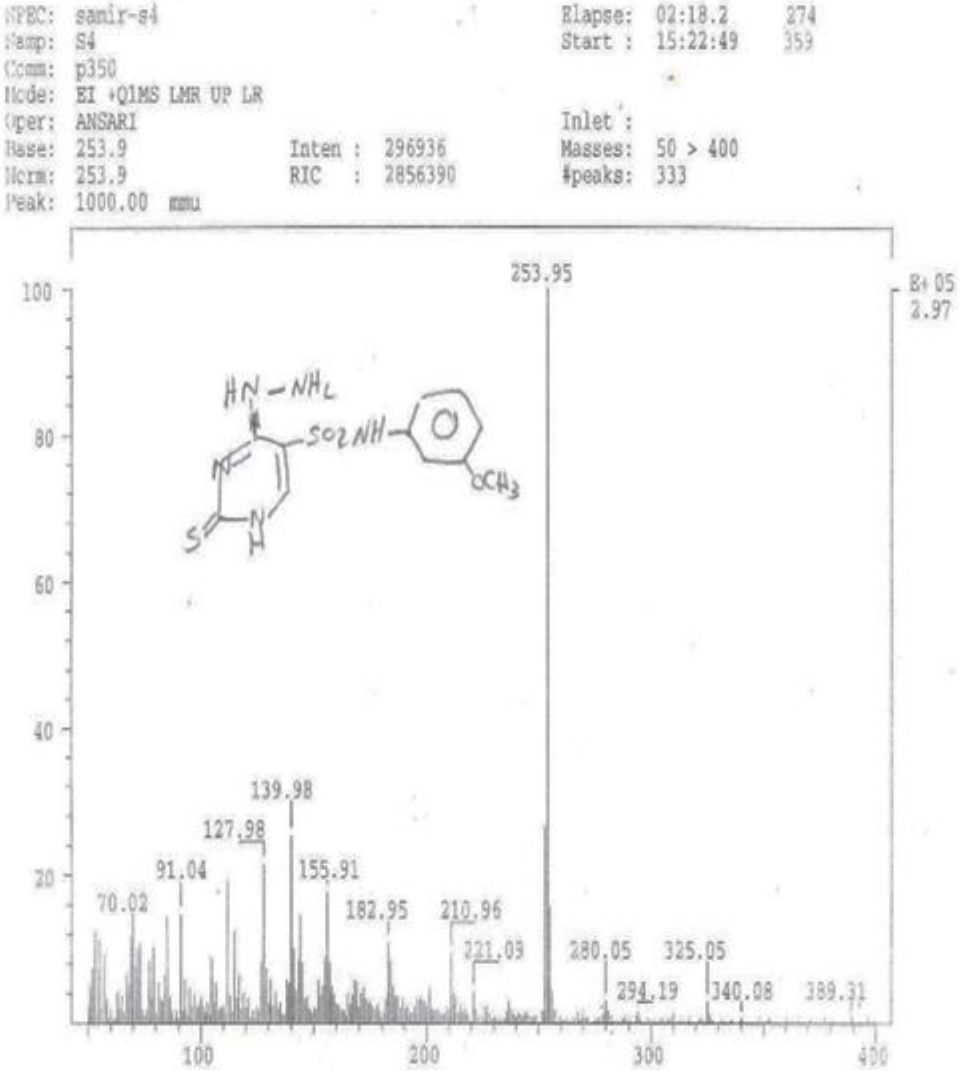

Figure 17. Low resolution mass spectrum of compound **6B**.

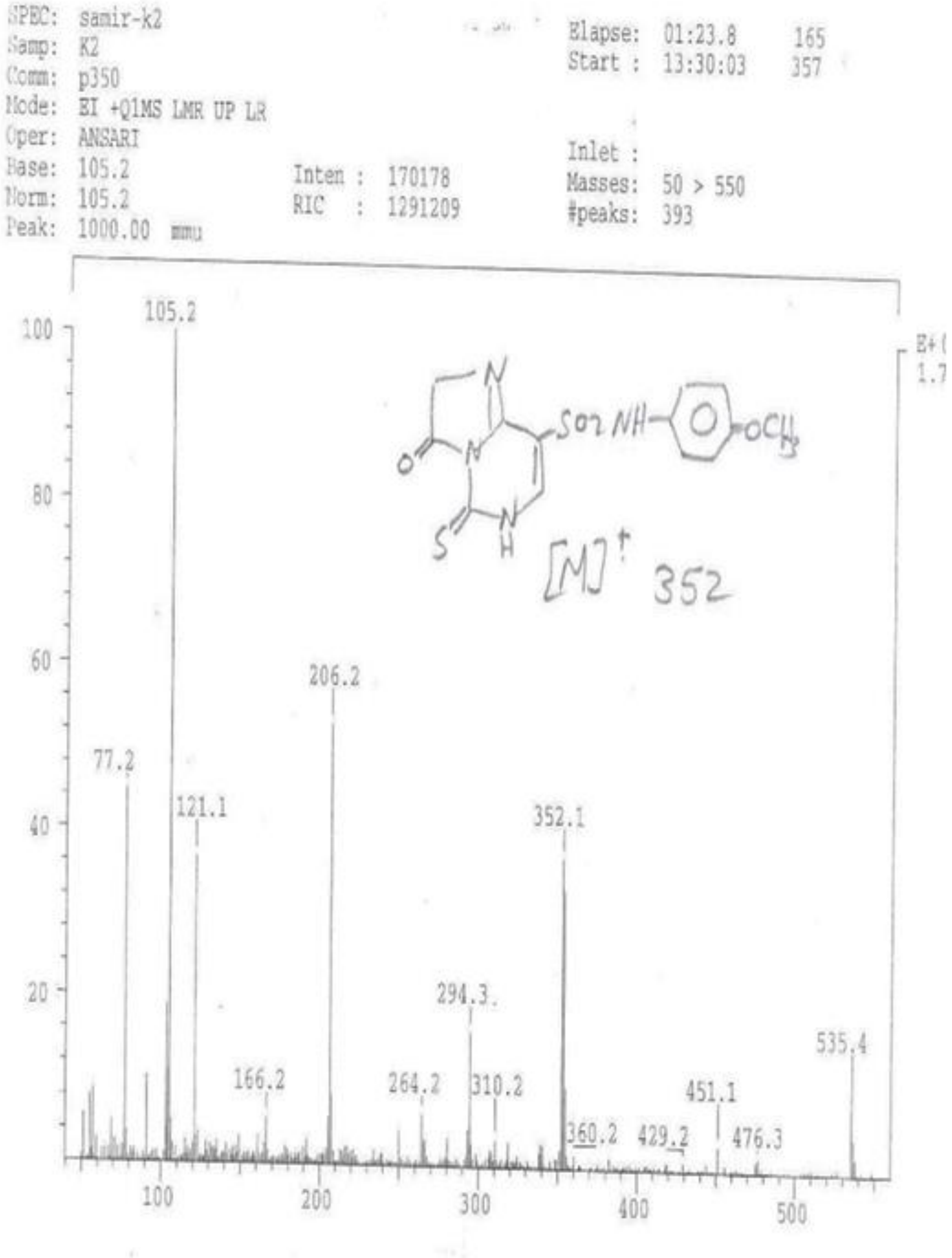

Figure 18. Low resolution mass spectrum of compound **7C**.

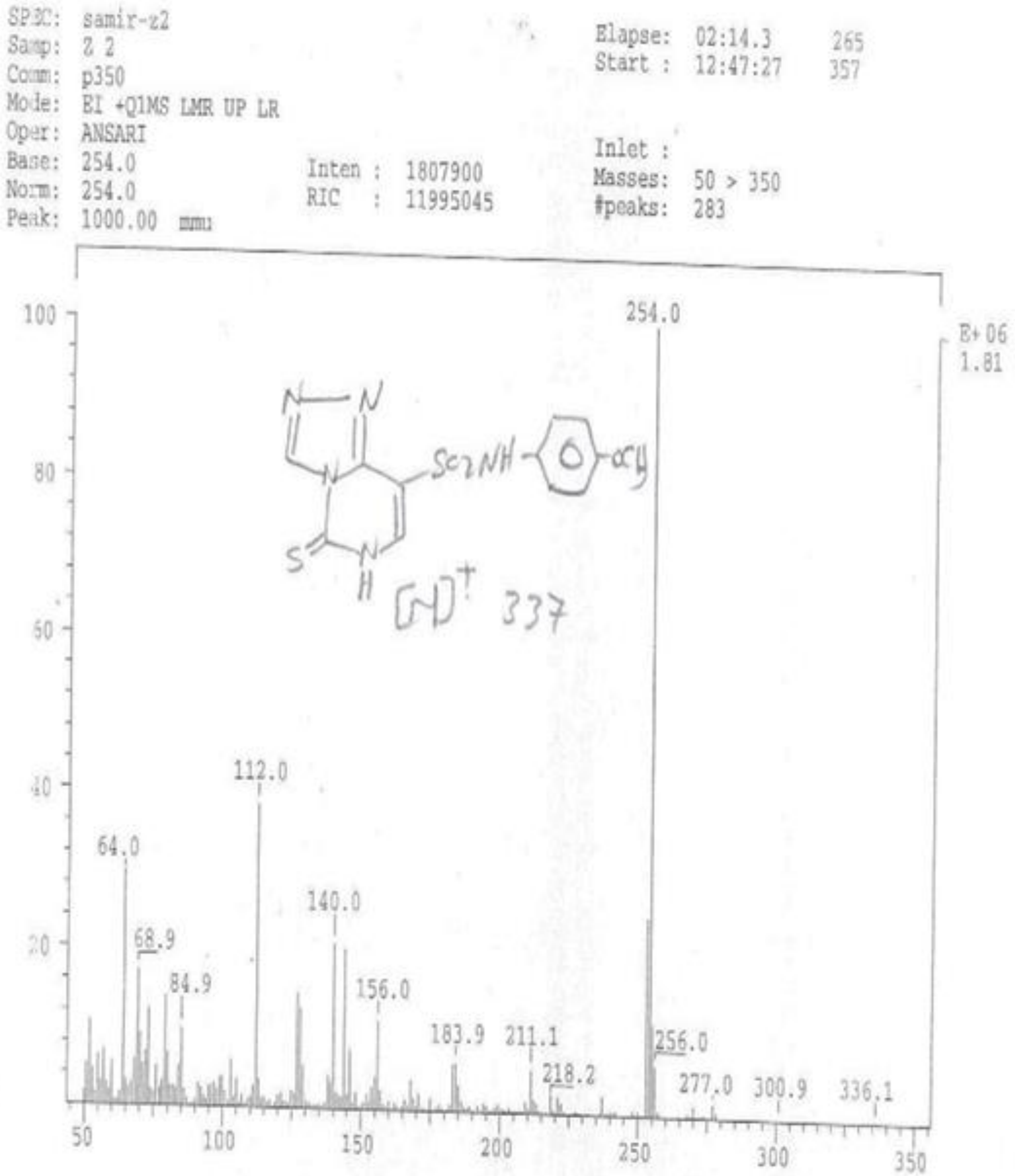

Figure 19. Low resolution mass spectrum of compound **9C**.

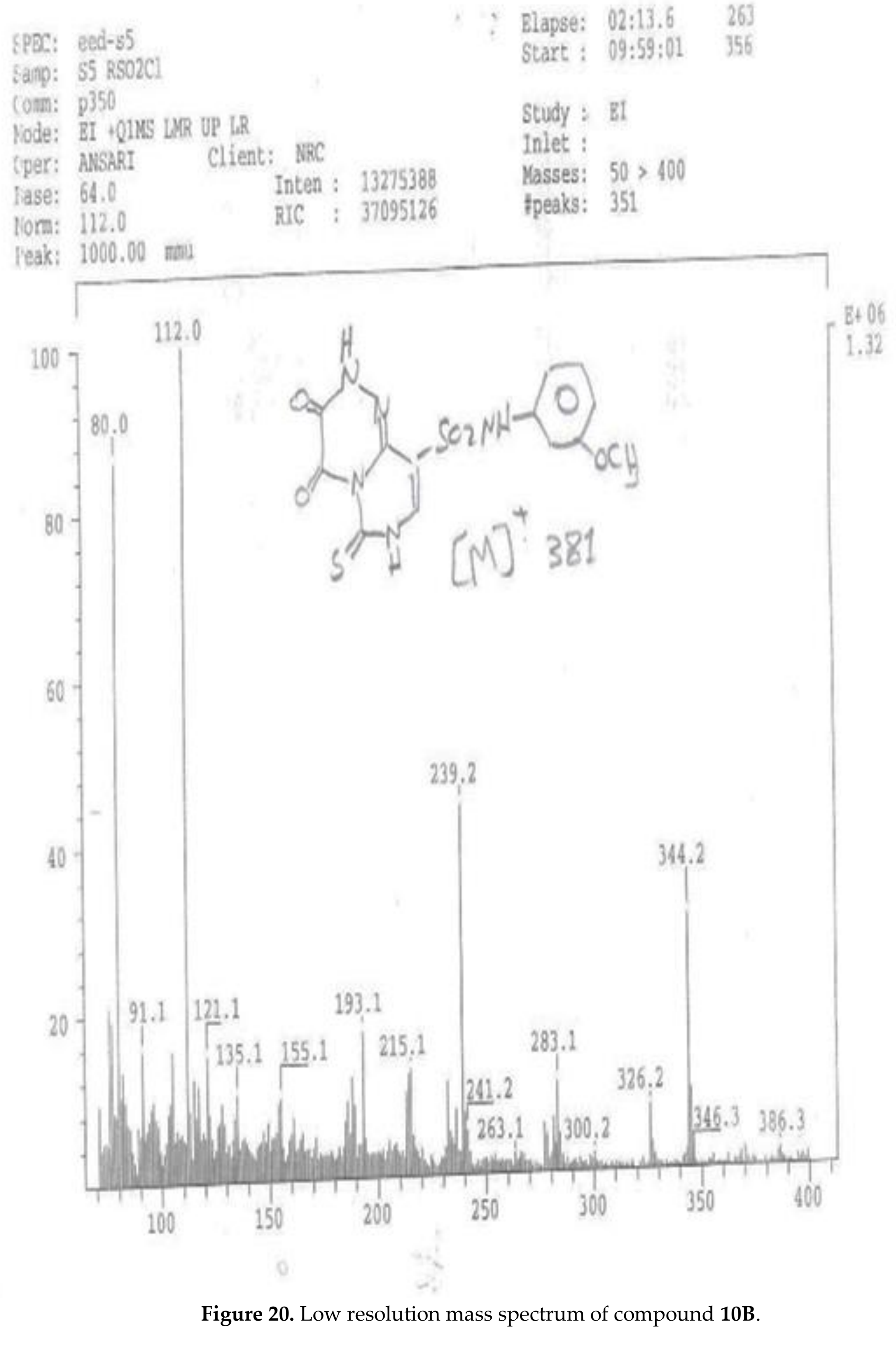

Figure 20. Low resolution mass spectrum of compound **10B**.

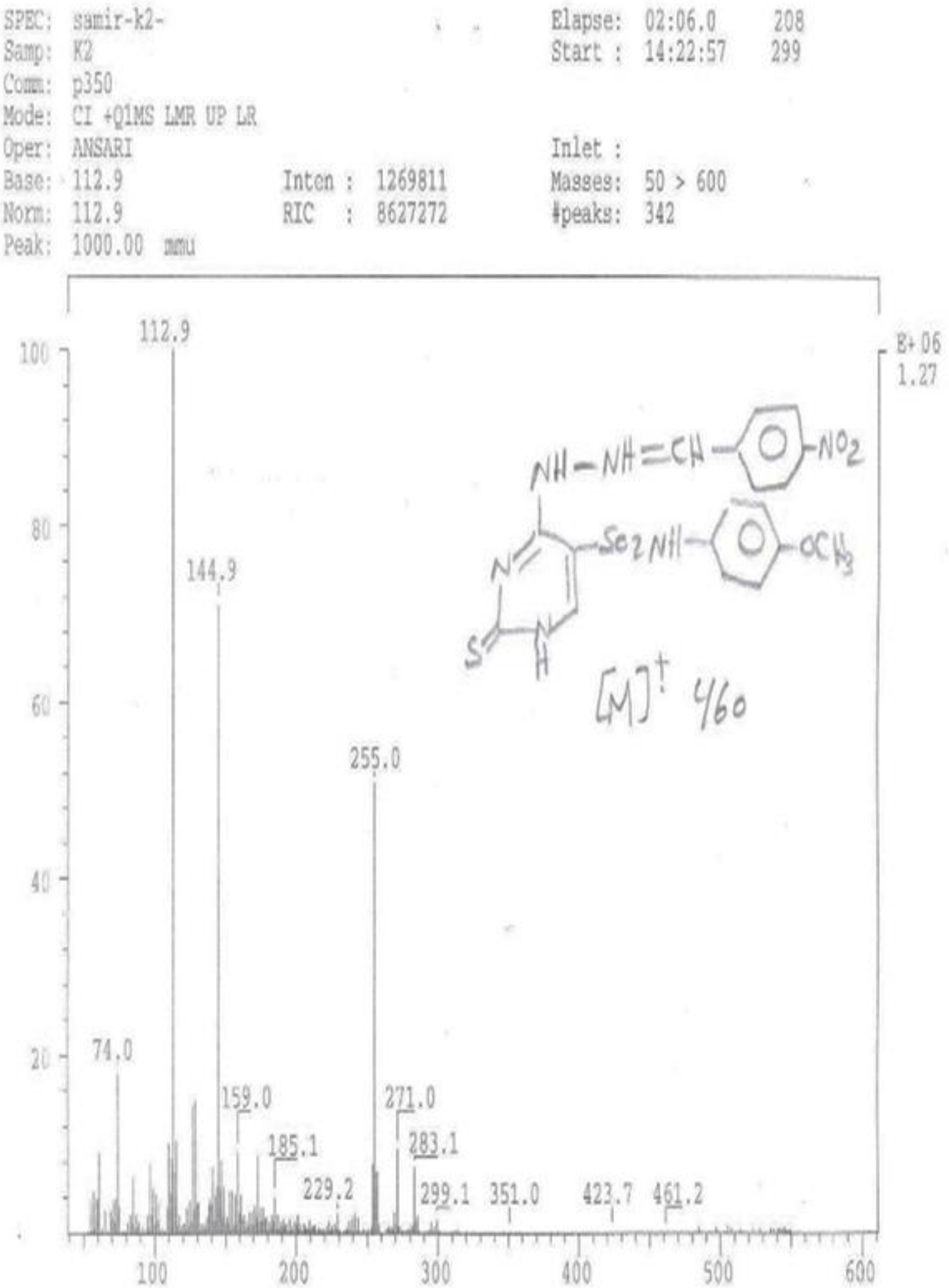

Figure 21. Low resolution mass spectrum of compound **11C**.

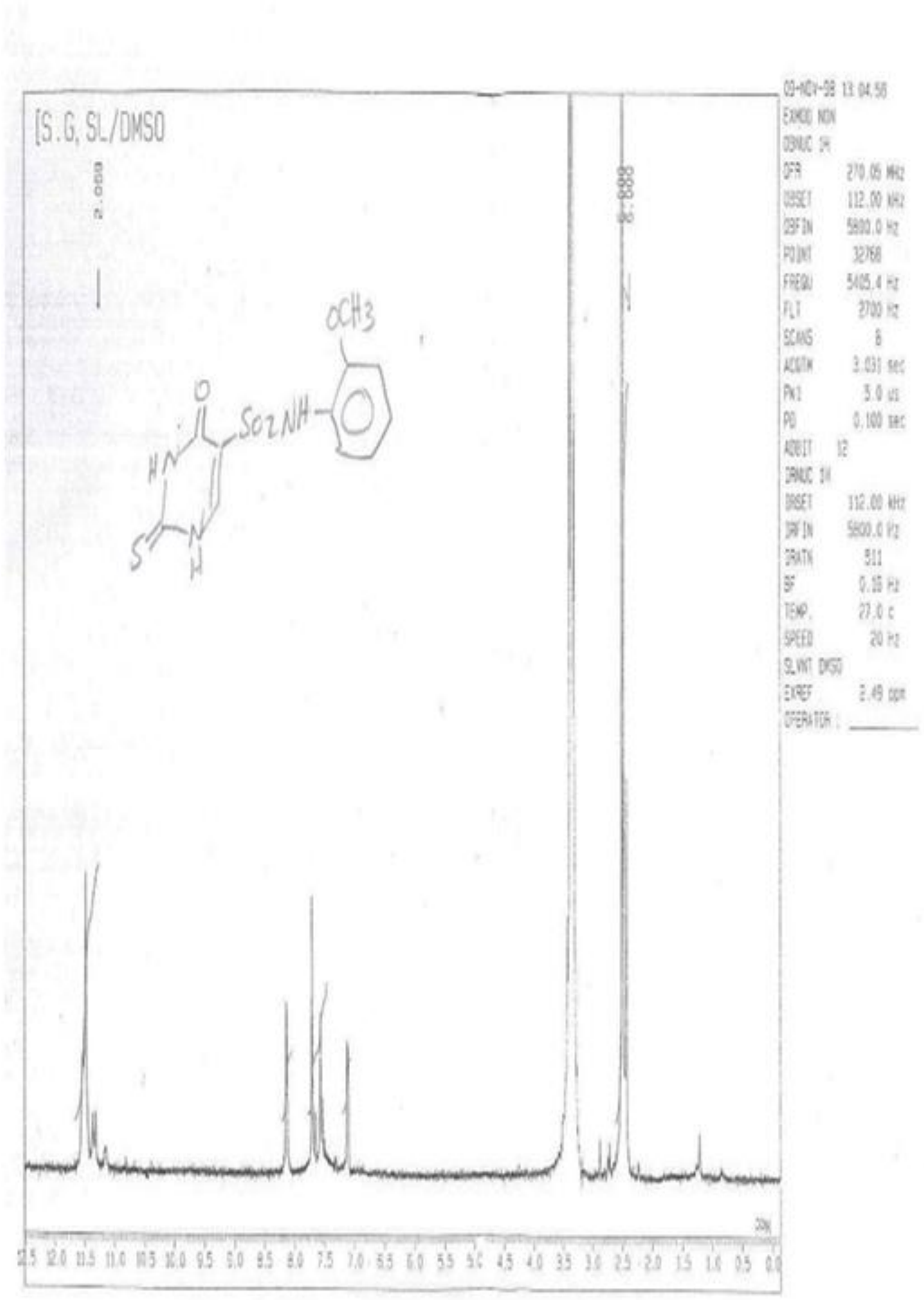

Figure 22. ^{1}H-NMR spectrum of compound **3A**.

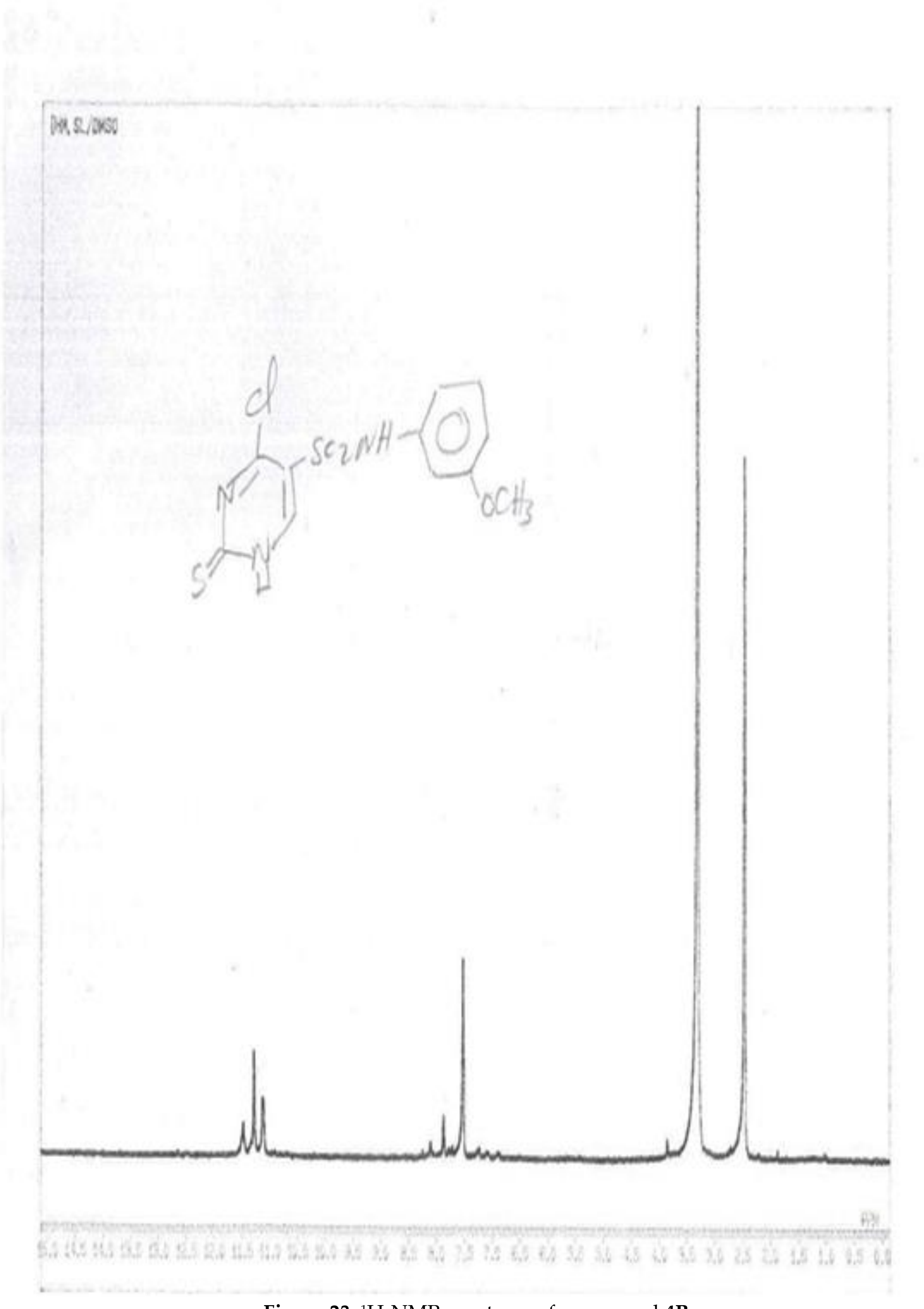

Figure 23. ^{1}H-NMR spectrum of compound **4B**.

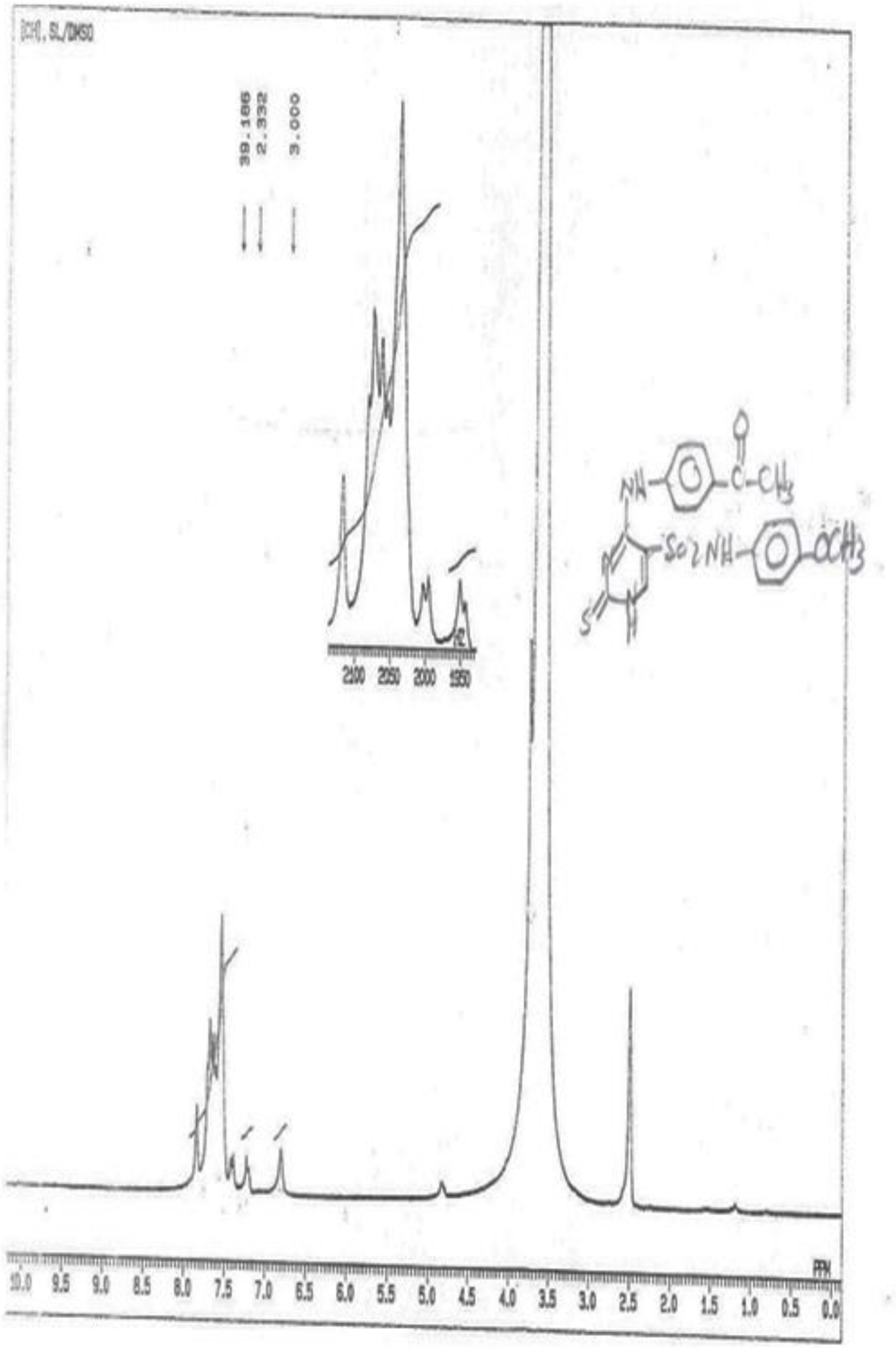

Figure 24. ^{1}H-NMR spectrum of compound **5C**.

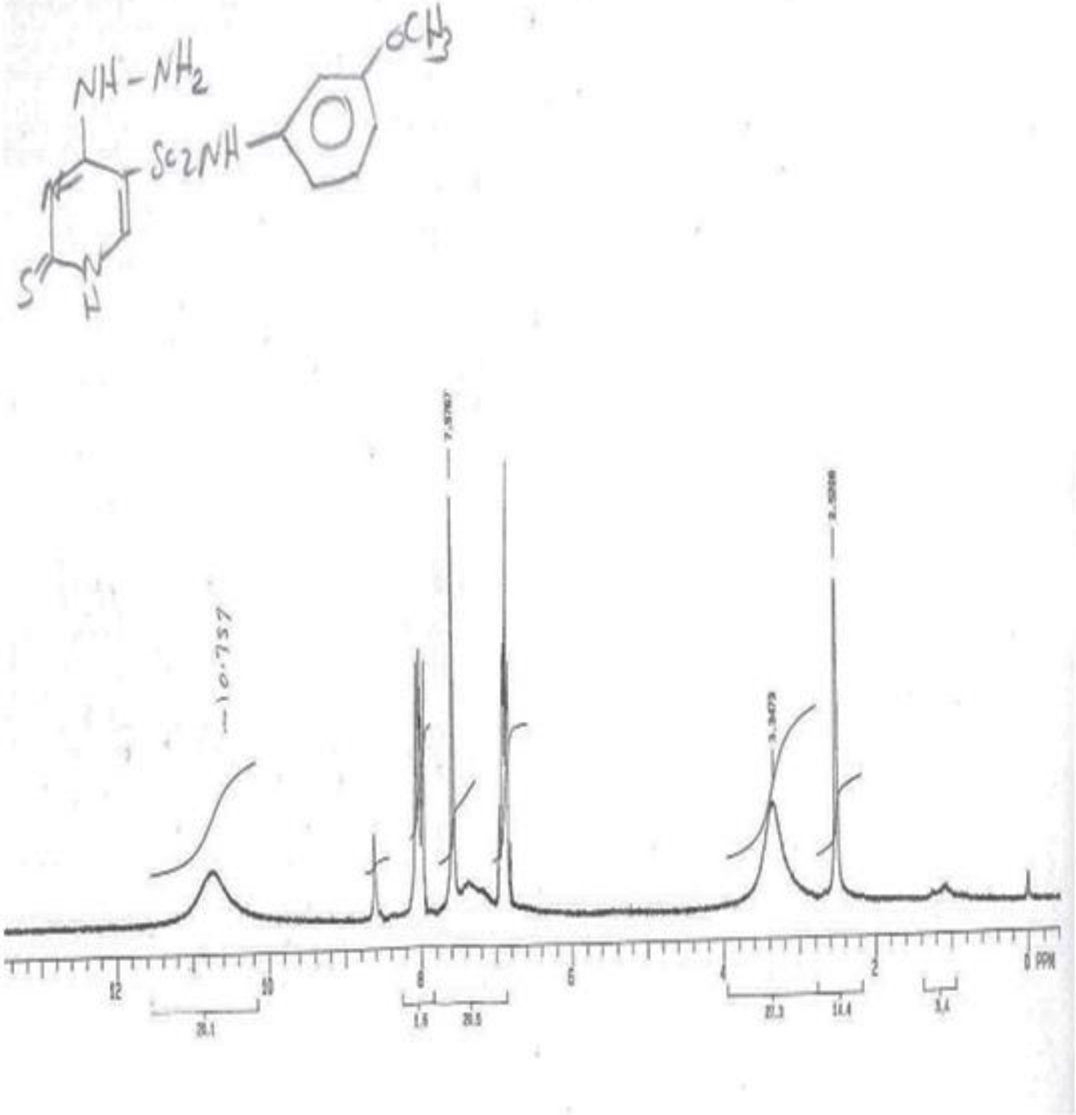

Figure 25. ^{1}H-NMR spectrum of compound **6B**.

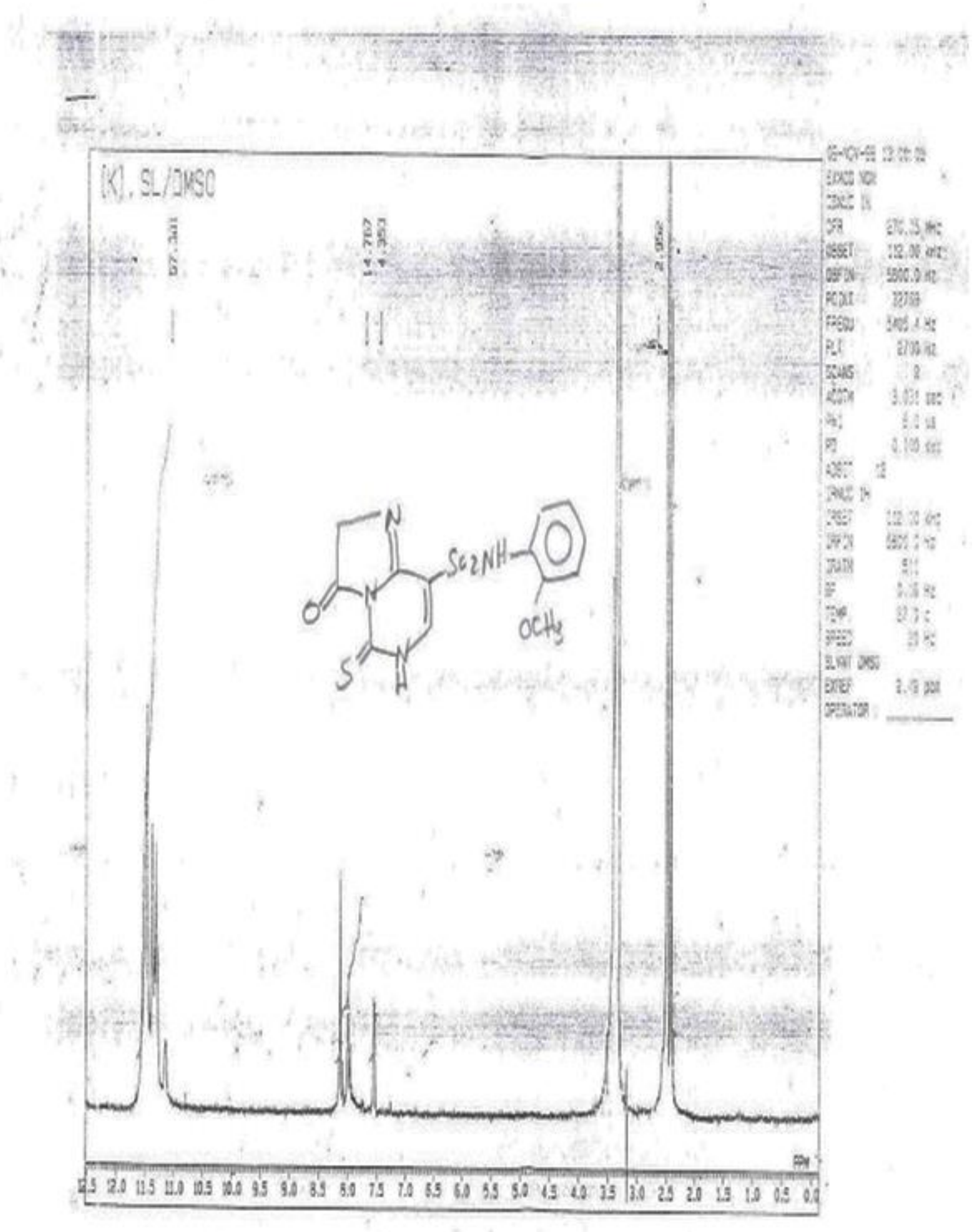

Figure 26. ^{1}H-NMR spectrum of compound **7A**.
